# Disentangling the Legacies of Climate and Management on Tree Growth

**DOI:** 10.1007/s10021-021-00650-8

**Published:** 2021-06-22

**Authors:** Laura Marqués, Drew M. P. Peltier, J. Julio Camarero, Miguel A. Zavala, Jaime Madrigal-González, Gabriel Sangüesa-Barreda, Kiona Ogle

**Affiliations:** 1 Department of Environmental Systems Science, Swiss Federal Institute of Technology (ETH Zürich), Universitätstrasse 2, 8092 Zürich, Switzerland; 2 Forest Ecology and Restoration Group, Department of Life Sciences, Universidad de Alcalá (UAH), Edificio Ciencias, Campus Universitario, 28871 Alcalá de Henares, Madrid, Spain; 3 School of Informatics, Computing, and Cyber Systems, Northern Arizona University, Flagstaff, Arizona 86011 USA; 4 Center for Ecosystem Science and Society, Northern Arizona University, Flagstaff, Arizona 86011 USA; 5 Instituto Pirenaico de Ecología, (IPE–CSIC), Avda. Montañana, 1005, 50192 Zaragoza, Spain; 6 Institute for Environmental Sciences, Climate Change Impacts and Risks in the Anthropocene, University of Geneva, 66 Boulevard Carl Vogt, 1205 Geneva, Switzerland; 7 Departamento de Biología Animal, Ecología, Edafología, Parasitología, Química agrícola, Universidad de Salamanca, Campus Miguel de Unamuno s/n, 37007 Salamanca, Spain; 8 EiFAB-iuFOR, Universidad de Valladolid, Campus Duques de Soria, 42004 Soria, Spain; 9 Department of Biological Sciences, Northern Arizona University, Flagstaff, Arizona 86011 USA

**Keywords:** *Abies alba*, Ecological memory, *Fagus sylvatica*, Forest management, Legacy effects, *Pinus sylvestris*, Spanish Pyrenees, Tree growth

## Abstract

Legacies of past climate conditions and historical management govern forest productivity and tree growth. Understanding how these processes interact and the timescales over which they influence tree growth is critical to assess forest vulnerability to climate change. Yet, few studies address this issue, likely because integrated long-term records of both growth and forest management are uncommon. We applied the stochastic antecedent modelling (SAM) framework to annual tree-ring widths from mixed forests to recover the ecological memory of tree growth. We quantified the effects of antecedent temperature and precipitation up to 4 years preceding the year of ring formation and integrated management effects with records of harvesting intensity from historical forest management archives. The SAM approach uncovered important time periods most influential to growth, typically the warmer and drier months or seasons, but variation among species and sites emerged. Silver fir responded primarily to past climate conditions (25–50 months prior to the year of ring formation), while European beech and Scots pine responded mostly to climate conditions during the year of ring formation and the previous year, although these responses varied among sites. Past management and climate interacted in such a way that harvesting promoted growth in young silver fir under wet and warm conditions and in old European beech under drier and cooler conditions. Our study shows that the ecological memory associated with climate legacies and historical forest management is species-specific and context-dependent, suggesting that both aspects are needed to properly evaluate forest functioning under climate change.

**Supplementary Information:**

The online version contains supplementary material available at 10.1007/s10021-021-00650-8.

## Highlights

- Multi-year climate legacies affect tree growth differently across species and sites.
- Critical time periods, typically the warmer and drier seasons, control tree growth.
- Historical forest management can buffer or predispose climate legacy effects.

## Introduction

Implicit in ecology is the idea that past states or antecedent conditions can influence the present or future of species dynamics (Margalef 1961; Warner and Chesson 1985). Past climate conditions are particularly important for predicting how forests may be affected by environmental perturbations occurring in tandem with climate change (Anderegg and others 2015; Kannenberg and others 2020; Ogle and others 2015; Schwalm and others 2017). Warming trends can modify the duration of the growing season, altering tree growth responses (Wolkovich and others 2012; Babst and others 2019), while drought events can reduce tree vitality and induce forest decline (Jump and others 2009; Allen and others 2010, 2015). Such past or antecedent climate can leave legacies or lagged effects on subsequent tree performance (Zweifel and others 2020; Monger and others 2015), affecting annual radial growth (Camarero and others 2018), long-term forest productivity (Liu and others 2018), and species coexistence (Johnstone and others 2016). Thus, climate legacies are defined as the persistent effects of antecedent climate conditions on current tree growth (Peltier and others 2016, 2018). Given species-specific responses to climatic variability (Babst and others 2013), it is important to understand how antecedent conditions affect the growth and productivity of different tree species and populations.

Forest productivity is also driven by management history (Noormets and others 2014). For example, past harvesting may be an essential driver of forest growth, setting the stage and conditions for forest dynamics over the long term (Paine and others 1998). Modifying stand density through management may reduce competition for resources (Linder 2000), improve post-drought resilience (McDowell and others 2006; D’Amato and others 2013; Sohn and others 2016), and consequently, facilitate forest adaptation to future climate change (Millar and others 2007; Marqués and others 2018). Furthermore, management decisions influence forest composition (Urbieta and others 2008), changing niche complementarity between coexisting tree species and modifying water- and resource-use efficiencies (González de Andrés and others 2017). Management practices could also lead to the selection of slow-growing trees that could become more vulnerable to drought-induced dieback (Reams and Huso 1990; Camarero and others 2011). The influence of human interventions (for example, management approaches) on ecosystems can thus have long-lasting legacies (Liu and others 2007). Therefore, understanding the causes of currently observed patterns and inferring forest vulnerability to climate change will likely require consideration of ecological memory, especially in terms of interactions between climate and management legacies.

The interactive influences of climate and management legacies are likely intense in mixed forests of the Spanish Pyrenees where several tree species reach their southern distributional limit in Europe (for example, silver fir), being therefore potentially highly sensitive to climate stress (Gazol and others 2015). Silver fir (*Abies alba* Mill.), European beech (*Fagus sylvatica* L.), and Scots pine (*Pinus sylvestris* L.) are dominant tree species in mixed Pyrenean mountain forests, where silver fir often forms mixed and pure stands in mesic and cool sites. These species differ in their climatic memory and sensitivity to climate variability (Gazol and others 2018) and have been subjected to different harvesting intensities over the last century as a function of their commercial value (De La Riva Fernández 1993). Silver fir and Scots pine are commonly managed for timber production, while European beech has been traditionally used for firewood and timber (Cabrera 2001). Silver fir is shade tolerant and highly sensitive to late-summer drought and cold prior winter conditions (Aussenac 2002; Camarero and others 2011; Lebourgeois and others 2013). Scots pine is shade intolerant, withstands cold conditions, but is vulnerable to spring and early summer drought stress (Camarero and others 2015a, b; Camarero and others 2018; Eilmann and Rigling 2012). European beech is shade tolerant and requires a humid atmosphere, tolerates cold winters, but can be particularly sensitive to late-spring frosts (Dittmar and others 2006) and warm and dry summer conditions (Gutiérrez 1988; Rozas and others 2015). Among these species, silver fir and European beech are expected to be the most shade tolerant, silver fir is expected to be the least drought tolerant, and Scots pine is expected to be the most frost tolerant (Niinemets and Valladares 2006). Thus, assessing the growth responses of these species to climate and management legacies is of special interest, particularly at the southern limits and near the dry edges of their distribution ranges.

Growth responses to climate have been traditionally evaluated using tree-ring width data, which provide one of the best proxies for quantifying long-term changes in tree radial growth and productivity (Fritts 1976). Tree-ring datasets often encompass varied spatial and temporal scales useful for understanding the long-term effects of climate and disturbances on tree growth (Babst and others 2014). However, tree-ring studies often do not consider information on past management, which may lead to an incomplete understanding of climate influences on tree growth (Bowman and others 2013) and biased scaling of tree- to stand-level responses (Zeide 2001; Pretzsch and Biber 2005). Therefore, forestry data obtained from historical management plans are important for evaluating climate-management interactions in forests (Pretzsch 2006; Madrigal-González and others 2015). To avoid potentially masking long-term signals, we analyzed raw tree-ring width data in the context of the stochastic antecedent modelling (SAM) approach (Ogle and others 2015; Peltier and others 2018). As implemented here, this approach simultaneously accounts for age, autoregressive, and covariate effects (for example, climate and/or management) on radial growth (Peltier and Ogle 2019a, 2019b). In this manner, the model allows for consideration of multiple sources of uncertainty that are essential to understanding the full range of factors governing tree growth (Biondi and Qeadan 2008).

In this study, we linked a network of tree-ring width data with historical forest management records from forests in the Spanish Pyrenees to simultaneously evaluate the effects of past climate and management on tree growth. We implemented the SAM approach within a hierarchical Bayesian framework for three focal species and three forest sites to address the following questions: (Q1) how do climate legacies affect tree growth, and what are the timescales over which these climate factors influence tree growth?; (Q2) how do these time-scales of influence translate annual climate records into antecedent climate effects?; and (Q3) what is the effect of historical management on tree growth, and how do management and climate interact to affect tree growth? We hypothesize that: (H1) related to Q1, multi-year legacy effects of climate influence tree growth, with longer timescales of influence (longer memory) at the more stressed sites; (H2) related to Q2, specific months of the year govern growth-climate relationships resulting in influential climate conditions that deviate from empirical annual climate records; and (H3) related to Q3, historical management interacts with climate legacies to affect tree growth, with the effects of harvesting differentially influencing the impacts of drought (for example, alleviating or aggravating) on tree growth depending on species and site conditions. Addressing these questions and hypotheses via the SAM approach can improve our understanding of the climate and management legacies imprinted on tree growth, with implications for managing forests under future climate change.

## Methods

### Study Area and Species

**Figure 1:**
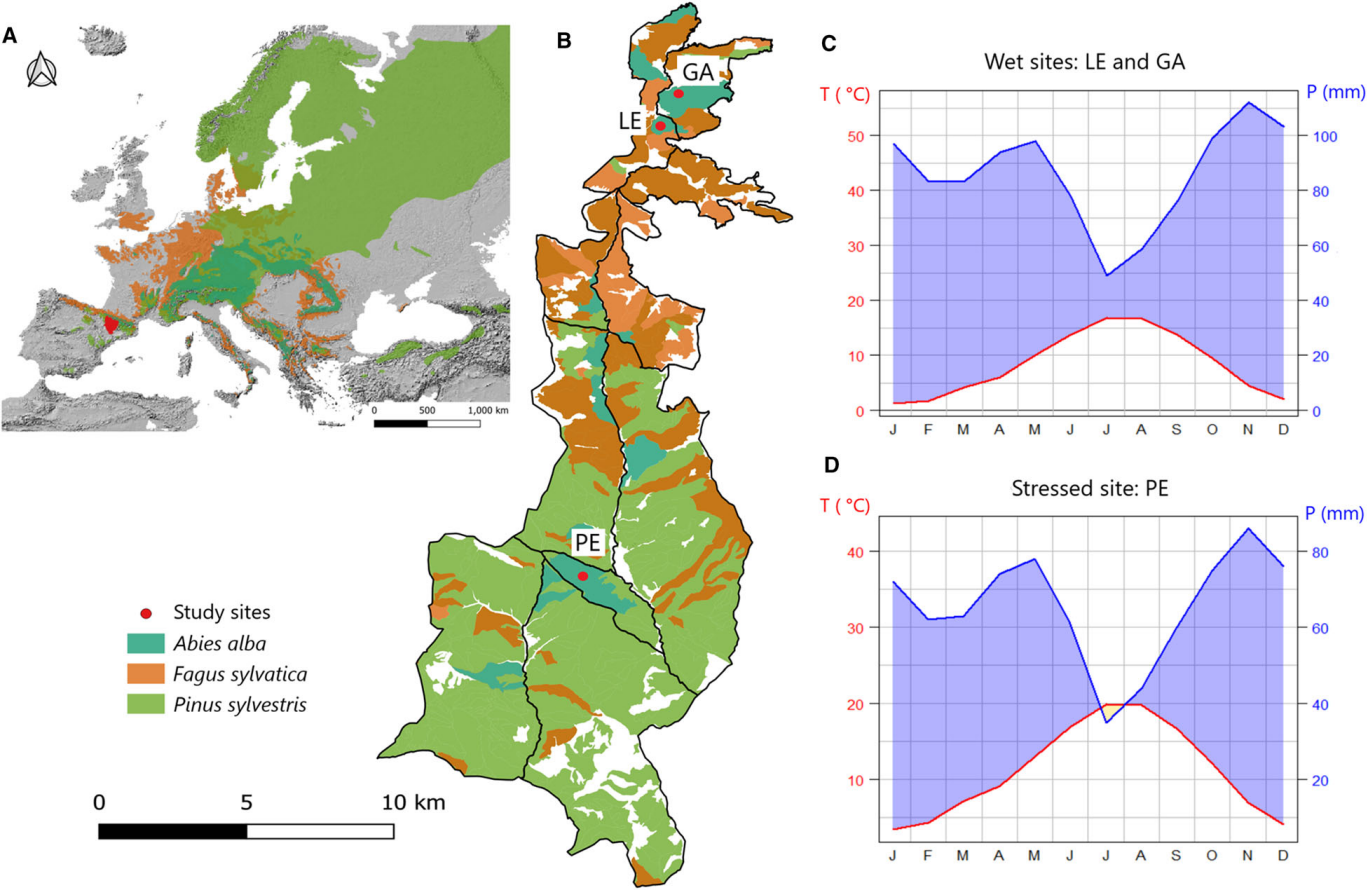
**A** Geographical location of Huesca province (Aragón, Spain) and geographic distributions of silver fir, Scots pine, and European beech forests in Europe. **B** Focal forested region divided into 10 subunits (black lines) for management purposes; the locations of the three study sites are indicated by red circles. **C**, **D** Climographs displaying data for monthly average temperature (T, °C, in red) and monthly average precipitation (P, mm, in blue) for **C** the wet-young (Las Eras, LE) and wet-old (Gamueta, GA) sites, and **D** the stressed site (Paco Ezpela, PE).

The study was conducted in formerly managed forest stands situated in the “Western Valleys” Natural Park (province of Huesca, Aragón), located in the west-central Spanish Pyrenees (1160–1465 m a.s.l.). The climate in the study area is continental with oceanic influence that leads to high precipitation in winter and a relatively narrow temperature range (for example, Figure 1). Across the study area, the mean annual temperature varies from 7.5 to 11.5 °C, and total annual rainfall ranges from 750 to 1500 mm, increasing with elevation (data obtained from local meteorological stations provided by the Spanish Meteorological Agency, AEMET). The geological substrates are mainly marls and limestones, which generate mollisols (USDA Soil Taxonomy, Soil Survey Staff 1999).

Within the region, we sampled three coexisting tree species (silver fir, Scots pine, and European beech) across three sites with different stand structure and climate conditions: Paco Ezpela (PE), Las Eras (LE), and Gamueta (GA) (Figure 1). Paco Ezpela (hereafter, “stressed site”) is characterized by warm and comparatively dry summers; it supports abundant and dominant silver fir trees (average age: 90 years), but with high levels of crown dieback (40% mean defoliation) and elevated mortality rates (42.1%) related to droughts in 1986, 1994–95, and 2012 (Camarero and others 2011, 2015a). Scots pine and European beech are also common at Paco Ezpela (average ages of ~ 95 and 50 years, respectively). Las Eras and Gamueta are cooler with more humid summers and colder winters. Both sites are located at a higher elevation, and Las Eras (hereafter, “wet-young site”) supports young, mixed forests with average ages of 50–60 years, while Gamueta (hereafter “wet-old site”) supports mature forests older than 100 years. In both wet sites, defoliation and mortality rates are very low, both in the range of 0–5% (Camarero and others 2011). Silver fir was sampled in all three study sites, while the other two species were sampled in two sites: European beech in the stressed and wet-old sites and Scots pine in the stressed and wet-young sites (see Table 1). Soils are of the loam and loamy sand types in all three sites, with soil pH varying from 6.0 to 6.5 (Supporting Information, Appendix S1). The percentage of sand is higher in the stressed site, while the percentage of silt is higher in the wet sites, which allows for higher water holding capacity.

**Table 1:** Geographical and topographical characteristics of the sampling sites, along with summaries of the number and attributes of the trees sampled.

Site	Elevation (m a.s.l)	Latitude (N)	Longitude (W)	Species	No. trees	No. cores	Basal area (m^2^ ha^−1^)	Mean tree-ring width (mm)	DBH (cm)	Age at 1.3 m (years)
Paco Ezpela (stressed)	1160	42º44’	0º49’	*A. alba*	36	56	15.2	1.4 ± 0.6	23.2 ± 8.8	90 ± 25
				*P. sylvestris*	22	40	10.5	1.1 ± 0.4	24.7 ± 6.9	95 ± 26
				*F. sylvatica*	48	77	2.2	0.7 ± 0.3	7.6 ± 4.0	51 ± 12
Las Eras (wet-young)	1310	42º52’	0º48’	*A. alba*	35	69	15.5	1.9 ± 0.9	23.4 ± 10.7	61 ± 9
				*P. sylvestris*	30	44	20.0	4.7 ± 1.7	29.1 ± 13.2	39 ± 40
Gamueta (wet-old)	1460	42º53’	0º48’	*A. alba*	34	46	55.8	1.7 ± 1.2	45.7 ± 15.2	138 ± 45
				*F. sylvatica*	26	34	8.2	1.8 ± 0.7	19.9 ± 7.3	70 ± 46

Values are means ± SD. DBH is the diameter measured at breast height (1.3 m). Species names correspond to *Abies alba* (silver fir), *Pinus sylvestris* (Scots pine), and *Fagus sylvatica* (European beech)

### Climate Data

We obtained monthly climate data (mean temperature and total precipitation) for the 1950–2016 period from the E-OBS v18.0 gridded dataset, which is derived from interpolation of the ECA&D (European Climate Assessment and Data) stations and provides homogenized and quality-controlled data at 0.25º spatial resolution (Haylock and others 2008). Both the wet-young and wet-old sites were associated with the same climate data since they occur within the same 0.25º spatial grid cell; the stressed site has its climate data.

### Dendrochronological Methods and Growth Assessments

During summer 2017, we sampled 22 to 48 trees per species-site combination, in a 0.5-ha sampling plot. We randomly selected trees to core within each plot to ensure that study trees were representative of the forest and that they spanned a range of sizes (ages) and levels of competition. We measured tree diameter at breast height (DBH, measured at 1.3 m) of each selected tree. Two cores were obtained per tree, perpendicular to the maximum slope and in opposite directions, using a Pressler increment borer. We also included tree-ring data from a previous sampling (2002), which were obtained via similar collection and measurement protocols. Both sampling periods resulted in a total of 231 trees being cored: 105 silver fir, 52 Scots pine, and 74 European beech (see Table 1).

Tree cores were prepared following standard dendrochronological methods (Fritts 1976) (See Appendices S2 and S3). Cores were air-dried, glued on wooden mounts, and polished on a sanding machine until the annual rings were clearly visible. Tree rings were visually cross-dated and widths measured to the nearest 0.01 mm using a binocular microscope and a LINTAB measuring device (Rinntech, Heidelberg, Germany) linked to a computer. Cross-dating of tree rings was checked using COFECHA (Holmes 1983). To estimate tree age at 1.3 m, pith-offset estimates were calculated by fitting a geometric pith locator to the innermost measured rings, and the estimated distance to the pith was added to the number of rings in the core (Applequist 1958).

### Forest Management Records

We digitized data from historical forest management archives provided by Spanish forestry services. These records usually contain long time series of forest structure and harvesting, which allowed us to examine the last century of stand dynamics (Madrigal-González and others 2017; Marqués and others 2018). In particular, we obtained data from the “Ansó-Fago” forest, which contains information compiled approximately every 10 years since 1928, with the most recent update in 2016. At the stand level, data include tree height, DBH, stand density, stand volume, and harvesting in terms of the number of trees and volume removed. Management information was generally recorded with annual resolution. The three focal tree species have been intensively managed in all sites since the early twentieth century (Cabrera 2001). The most frequently used method of harvesting was diameter limit or selection thinning, which primarily removed dominant and fast-growing trees (in the 20–25 cm DBH class; Aunós and Blanco 2006). For administrative purposes, the forest was subdivided into ten subunits (“cuarteles” in Spanish; mean size = 1,395 ha; see solid lines of the map in Figure 1), each of which was further partitioned into five stands (“tramos” in Spanish; mean size = 206 ha). Each sampling plot was located in one of the stands (53.42, 139.66, and 108.89 ha at the stressed, wet-young, and wet-old sites, respectively). For each of these stands, stand density was assessed by counting all individuals of a given species, and stand volume was estimated from DBH and height measurements of each tree considering allometric coefficients. We calculated the variable “harvesting intensity” (*HI*) as the ratio of the number of trees removed per year relative to the stand density of the three tree species obtained from the historical forest management plans. To assure that past management effects are correctly captured, we also repeated our analyses (see section below) with harvesting intensity defined as the percentage of wood volume removed from the stands (Storch and others 2019). Values of stand density (trees per ha), stand volume (m^3^ per ha), harvested trees, and harvested volume from each stand are provided for each species and site in Appendix S4.

### Model Description

We used the stochastic antecedent modeling (SAM) framework (Ogle and others 2015) to quantify the effects of past climate and management on tree growth. The SAM approach allowed us to estimate the relative importance of current and antecedent climate variables on annual tree growth, the potential periods of greatest influence, and the variation in the relative importance of conditions occurring at different times into the past. In addition, we simultaneously included historical forest management (that is, harvesting intensity) in the model.

We applied the SAM approach to raw tree-ring width data for silver fir, Scots pine, and European beech to explore the legacy effects of monthly climate up to four years prior to ring formation. We chose a five-year period (current year and up to four years prior) because this is the maximum mean drought recovery time suggested by Anderegg and others (2015), and it has been successfully used in other applications of the SAM model to tree-ring widths (Peltier and others 2016, 2018). Longer lags could be considered, but this would increase model complexity and computational requirements. We simultaneously accounted for the uncertainty associated with biological growth trends by including age and autoregressive effects (Tingley and others 2012) within the modelling framework.

Measured ring width (*r*, mm) provided a direct index of radial tree growth, which we log-transformed, yielding *G* = log(*r* + 1), to better meet the assumption of normally distributed errors; we used *r* + 1 since there are several instances where *r* = 0 (for example, missing rings). The observed (data) and expected (mean) log-scale ring width varied at the level of year *t* and core *c*. The likelihood of the observed data, with mean *μ* and variance *σ*^2^, is thus defined as:

1 $$ \begin{array}{*{20}c} {G_{t,c} \sim {\text{Normal}}\left( {\mu_{t,c} ,\sigma^{2} } \right)} \\ \end{array} $$

The expected (or predicted mean) growth, *μ*, was modelled as a function of tree age (Age) associated with the ring formed in year *t*, antecedent precipitation (*P*^*ant*^), antecedent temperature (*T*^*ant*^), harvesting intensity (*HI*), and the previous year’s growth (*G*_*t*−1_), with the latter representing a first-order autoregressive effect. We used tree age rather than tree size following standard age-detrending approaches and prior applications of the SAM model (for example, Ogle and others 2015 and Peltier and others 2016); the goal was to simply account for tree age, thus allowing more accurate estimates of the climate and *HI* effects. Thus, the mean model is given by:

2 $$ \begin{aligned} \mu_{t,c} & = \alpha_{1,c} + \alpha_{2,c} \cdot {\text{Age}}_{t,c} + \alpha_{3,c} \cdot P_{t}^{{{ant}}} + \alpha_{4,c} \cdot T_{t}^{{{ant}}} + \alpha_{5,c} \cdot P_{t}^{{{ant}}} \cdot T_{t}^{{{ant}}} \\ & \quad + \alpha_{6,c} \cdot G_{t - 1,c} + \alpha_{7,c} \cdot HI_{t} + \alpha_{8,c} \cdot HI_{t} \cdot P_{t}^{{{ant}}} + \alpha_{9,c} \cdot HI_{t} \cdot T_{t}^{{{ant}}} \\ \end{aligned} $$

All five covariates—Age, *P*^*ant*^, *T*^*ant*^, *G*_*t*−1_, and *HI*—in Eq. (2) were centered around the site- and species-level sample means representative of the target time period (1950–2016). Thus, the core-level intercept (*α*_1_)—and associated tree- and site-level intercept [see Eq. (4)]—represents the predicted or base-line growth at the average age, prior year’s growth, climate, and management conditions. The other coefficients depict the age effect (*α*_2_), the main effects of antecedent precipitation and temperature (*α*_3_ and *α*_4_, respectively), their corresponding interaction effect (*α*_5_), the autoregressive effect (*α*_6_), the main effect of harvesting intensity (*α*_7_), and its corresponding interaction with antecedent precipitation and temperature (*α*_8_ and *α*_9_, respectively). We explored other moisture indices (that is, the ratio of precipitation to potential evapotranspiration), but the resultant models led to computational challenges or produced worse model fits than the models including temperature, precipitation, and their interaction. The strength of this approach is its simplicity, where responses to precipitation and temperature are directly interpretable without reference to assumptions inherent to other moisture indices.

The SAM framework calculates the antecedent climate variables as a weighted average of monthly precipitation or temperature over a 5-year (60 months) period. Here, each antecedent climate variable, $X_{t}^{{{\text{ant}}}}$, is defined as:

3 $$ X_{t}^{{{\text{ant}}}} = \mathop \sum \limits_{y = 0}^{4} \mathop \sum \limits_{m = 1}^{12} X_{t - y,m} \cdot w_{y,m,v} $$

where *X* = *P* or *T* for precipitation or temperature, respectively; *X*_*t*−*y*,*m*_ denotes the climate variable for month *m* (*m* = 1, 2,…, 12) and for *y* years into the past (*y* = 0, 1,…, 4 for the year of ring formation, prior year, …, 4 years prior) relative to year *t*; and *w*_*y*,*m*,*v*_ denotes the antecedent importance weight, estimated for year *y* into the past, month *m*, and variable *v* (*v* = 1 for precipitation and *v* = 2 for temperature). Precipitation and temperature conditions occurring after the cessation of growth cannot affect ring width during the same year, and thus weights for October, November, and December (*m* = 10, 11, 12) of the current year (*y* = 0) are fixed at zero for both climate variables. The temporal resolution of the weights, *w*_*y*,*m*,*v*_, declines with increasing time into the past (1 month or blocks of 2, 3, or 4 months); see Peltier and others (2018) for further details.

We computed annual weights for each calendar year into the past by summing the monthly weights over all months within a given year. We also computed the cumulative monthly weights, akin to a cumulative probability, by summing the monthly weights, *w*_*y*,*m*,*v*_, over past years (*y*) and months (*m*). The degree to which the cumulative monthly weights change over time (indexed by both *y* and *m*) provides information about the timescales of influence. In particular, we defined two thresholds at *c* = 0.5 and *c* = 0.9 cumulative monthly weights, giving *M*_50_ and *M*_90_, which are the climate memory lengths (months into the past) when the cumulative weights reach 50% and 90%, respectively. In addition, to evaluate the influence of antecedent climate on tree growth, we computed the uncentered antecedent climate variables, enabling direct comparison against the average annual climate values obtained directly from the climate records.

We implemented the above model in a hierarchical Bayesian framework. In doing so, we assumed hierarchical priors for the core-level parameters, *α*_*k*,*c*_ [see Eq. (2)], with global means, *μ*_*α*_, and variances, *σ*_*α*_^2^, such that, for coefficient index *k* (*k* = 1, 2, …, 9) and core *c*:

4 $$ \alpha_{k,c} \sim {\text{Normal}}\left( {\mu_{{\alpha_{k} }} ,\sigma_{{\alpha_{k} }}^{2} } \right) $$

The prior for the age effect (*α*_2_,_*c*_) was truncated to negative values to be consistent with the negative exponential curve typically used to model the effect of age (Fritts 1976). All other trends were considered to be ecological variation and thus preserved by this approach.

Finally, we assigned relatively non-informative priors to the remaining, global parameters, including diffuse normal priors for the global effects, all $\mu_{{\alpha_{k} }}$ in Eq. (4); wide uniform priors for all standard deviation terms, *σ* in Eq. (1) and all $\sigma_{{\alpha_{k} }}$ in Eq. (4); and a relatively non-informative Dirichlet prior to the vector of monthly importance weights, ***w***_*v*_, which is composed of all *w*_*y*,*m*,*v*_ for all *y* and *m*; see Eq. (3). The latter ensures that all monthly weights, *w*_*y*,*m*,*v*_, are between 0 and 1 and are constrained to sum to one over all *y* and *m*, for each variable *v*.

### Model Implementation

All analyses were performed in R (version 3.4.3; R Core Team 2018). Tree-ring width data were processed via the *dplR* package (Bunn 2008). The SAM model was coded and implemented in JAGS 4.3.0 (Plummer 2003) using the *rjags* package in R (Plummer 2018). The SAM model was implemented separately for each species-site combination, and three parallel Markov chain Monte Carlo (MCMC) sequences (or chains) were simulated to sample from the posterior distribution of all model parameters. We monitored all parameters of interest, with a specific focus on the species-site-level coefficients (*μ*_*α*_), the importance weights (monthly weights, *w*, along with the cumulative and annual weights), and the timeseries of the predicted antecedent climate variables, *T*^*ant*^ and *P*^*ant*^. Convergence of the MCMC sequences was evaluated using the potential scale reduction factor (Gelman and Rubin 1992). After an initial burn-in period (> 10,000 iterations), the sequences were run for more than 300,000 iterations and were thinned every 10th iteration to reduce within sequence autocorrelation and storage requirements. A posterior sample size greater than 10,000 was used for computing posterior summary statistics. Model code and a list of all R packages used are included in Appendix S5. MCMC trace plots for each species and site are shown in Appendix S6. To assess the fit of the above SAM model to the data, regressions of predicted (replicated; as per Gelman and others 2013) versus observed growth data (*G*) were performed for each site-species combination to calculate coefficients of determination (*R*^2^; Appendix S7).

## Results

Here we report the results for the model described herein [for example, Eq. (2)] that includes harvesting, *HI*, as the ratio of the number of trees removed per year relative to the stand density of the three tree species. A subset of results from models with *HI* defined in terms of volume removed from the stand is shown in Appendix S10.

### Model Performance

Model fit (*R*^2^) varied between 0.73–0.94 across all species and sites (see Appendix S7). Species-specific *R*^2^ values were higher for silver fir (*R*^2^ = 0.89–0.94) and lower for European beech (*R*^2^ = 0.73–0.75). Model fits were more variable among sites for Scots pine, with *R*^2^ = 0.77 in the stressed site and *R*^2^ = 0.94 in the wet-young site. The autoregressive effect only accounted for 0.5–6% of the overall model fit, pointing to the general importance of antecedent climate and/or management.

### Effects of Past Climate and Harvesting on Tree Growth

**Figure 2:**
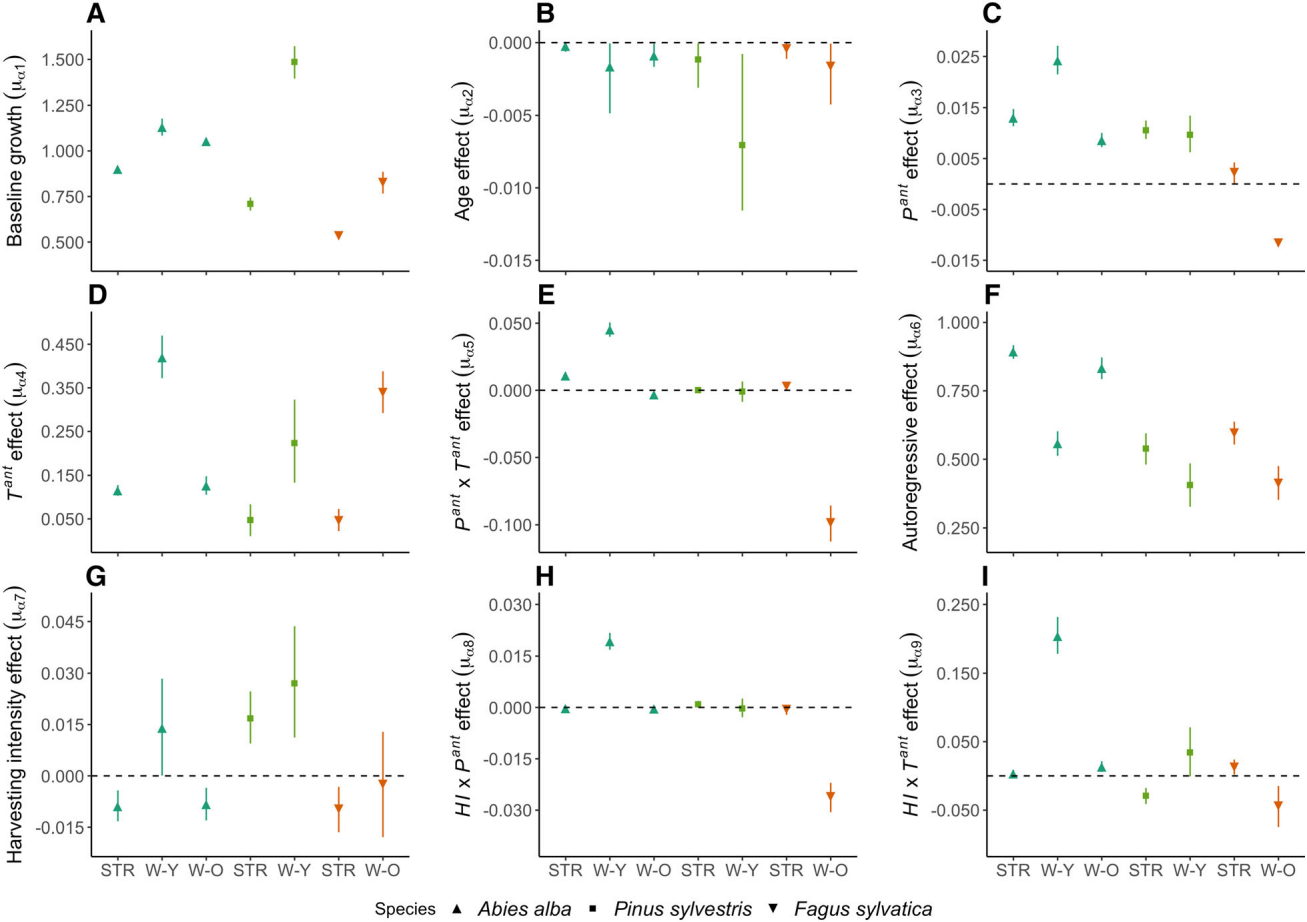
Posterior means (symbols) and 95% Bayesian credible intervals (CIs, whiskers) for the population-level (species-site-level) regression coefficients [*μ*_*α*_ terms, see Eq. (4)] describing: **A** log-scale baseline growth (intercept) at average conditions, and the effects of **B** age, **C** antecedent precipitation (*P*^*ant*^), **D** antecedent temperature (*T*^*ant*^), **E** the *P*^*ant*^ × *T*^*ant*^ interaction, **F** prior ring width (that is, autoregressive effect), **G** harvesting intensity (*HI*), **H** the *HI* × *P*^*ant*^ interaction, and **I** the *HI* × *T*^*ant*^ interaction. Effects with CIs that do not overlap zero (dotted horizontal line) are considered significant. Coefficient estimates are shown for each species from left to right: *Abies alba* (triangles); *Pinus sylvestris* (squares); *Fagus sylvatica* (inverted triangles); and sites: stressed (STR), wet-young (W-Y), wet-old (W-O).

Baseline log-scale growth, $\mu_{{\alpha_{1} }}$ (population-level intercept), was tightly constrained (narrow CIs) and differed significantly among all species and sites (Figure 2A). The lowest baseline growth was predicted for European beech at the stressed site (equivalent to ca. 0.7 mm), while the highest was estimated for Scots pine at the wet-young site (ca. 3.44 mm). The age effects for most species and sites were estimated to be very close to zero, indicating that ring widths were nearly independent of age over the period considered in the analysis (Figure 2B and Appendix S8). Scots pine in the wet-young site, however, exhibited a stronger negative age-growth trend; trees sampled at this site were much younger than at the other sites (Table 1). The autoregressive term, $\mu_{{\alpha_{6} }}$, was significantly positive for all species and sites (Figure 2F).

The effects of antecedent climate (*P*^*ant*^, *T*^*ant*^, and their interaction) were generally consistent in their direction (for example, positive or negative) across sites and species, with a few exceptions. The main effects of antecedent precipitation (*P*^*ant*^), $\mu_{{\alpha_{3} }}$, were significantly positive for all species and sites, except for European beech at the wet-old site, where the *P*^*ant*^ main effect was negative (Figure 2C). The main effects of antecedent temperature (*T*^*ant*^), $\mu_{{\alpha_{4} }}$, were significantly positive for all species and sites, with the largest positive effect occurring for silver fir at the wet-young site (Figure 2D). The *P*^*ant*^ × *T*^*ant*^ interaction effect, $\mu_{{\alpha_{5} }}$, was significantly positive for silver fir at the stressed and young-wet sites and European beech at the stressed site, but this interaction was negative for silver fir and European beech at the old-wet site (Figure 2E).

The main effect of harvesting intensity (*HI*), $\mu_{{\alpha_{7} }}$, was significantly positive for silver fir at the wet-young site and Scots pine at the stressed and wet-young sites (Figure 2G). However, the *HI* main effect was negative for silver fir at the stressed and wet-old sites as well as for European beech at the stressed site. Silver fir at the wet-young site and European beech at the wet-old site stood out as having strongly positive and negative, respectively, *HI* × *P*^*ant*^ interaction effects, $\mu_{{\alpha_{8} }}$; this interaction was non-significant or only marginally significant for all other species sites (Figure 2H). The *HI* × *T*^*ant*^ interaction effect, $\mu_{{\alpha_{9} }}$, was significantly positive for silver fir at the wet-young and wet-old sites and for European beech at the stressed site, but significantly negative for Scots pine at the stressed site and for European beech at the wet-old site (Figure 2I).

Interpretation of how *P*^*ant*^ and *T*^*ant*^ influence growth requires a more detailed consideration of the main effects and interaction terms (Neter and others 1996). For example, the net sensitivity of tree growth [*G*, see Eq. (1)] to changes in *P*^*ant*^ is given by d*G*/d*P*^*ant*^ = *α*_3_ + *α*_5_*·T*^*ant*^ + *α*_8_*·HI*, based on Eq. (2), where the *α*’s correspond to the core-level [*α*’s in Eq. (2)] or population-level [*μ*_*α*_’s in Eq. (4)] main effects (for example, *α*_3_) and interaction effects (for example, *α*_5_ and *α*_8_). When using population-level coefficients (Figure 2), growth of silver fir at the wet-young site and of European beech at the wet-old site are being highly sensitive to antecedent climate relative to the other populations, but these two populations exhibit divergent behavior. In general, under low values of *T*^*ant*^ and *HI*, higher *P*^*ant*^ is predicted to reduce growth of silver fir at the wet-young site (Appendix S9a). However, growth in this population is stimulated by higher *P*^*ant*^, especially under high values of *HI* and *T*^*ant*^ (Appendix S9b). European beech at the wet-old site is predicted to respond in the opposite fashion: growth is generally reduced by higher *P*^*ant*^, especially under high *HI* in combination with high *T*^*ant*^, and growth is only expected to be stimulated by increased *P*^*ant*^ when both *HI* and *T*^*ant*^ are low (Appendix S9a and S9b). The net sensitivities to *T*^*ant*^ (d*G*/d*T*^*ant*^) indicate that higher *T*^*ant*^ stimulates growth in silver fir at the wet-young site, except when *P*^*ant*^ and *HI* are low, in which case growth is reduced by higher *T*^*ant*^ (Appendix S9c). Again, European beech at the wet-old site exhibits a different pattern: under high *P*^*ant*^, higher *T*^*ant*^ reduces growth, whereas growth is stimulated by higher *T*^*ant*^ when *P*^*ant*^ is low, regardless of the values of *HI* (Appendix S9c and S9d).

### Climatic Temporal Pattern and Memory Length

**Figure 3:**
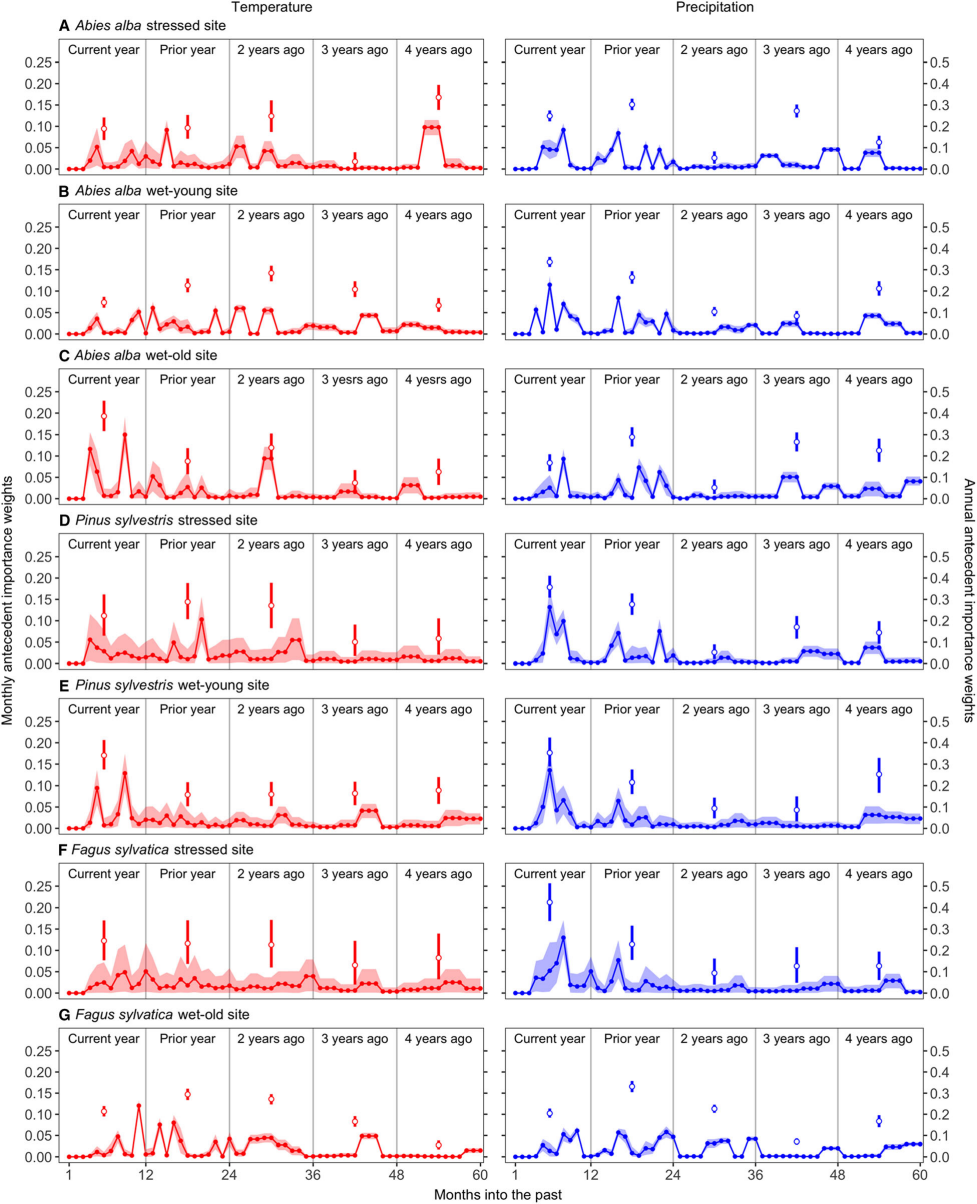
Monthly and yearly antecedent importance weights for both antecedent climate variables (left column = *T*^*ant*^; right column = *P*^*ant*^) for all species and sites (rows). The months and years are ordered from most to least recent such that, for example, month *m* = 12 and year *y* = 0 corresponds to December of the current year (‘months into the past’ = 1) and month *m* = 1 and year *y* = 4 corresponds to January 4 years prior to the current year (‘months into the past’ = 60). Filled circles connected by lines are the posterior means of the monthly weights, and the shaded area represents the 95% Bayesian credible intervals (CIs). Empty circles are the posterior means of the yearly weights (sum of monthly weights within a given year) and whiskers are the corresponding 95% CIs.

Given the importance of antecedent climate (Figure 2C–E), we evaluated the antecedent importance weights, *w*_*y,m,v*_, to explore differences in climate legacies among species and sites. Based on the annual importance weights, temperature and precipitation occurring during the year of ring formation (*t* = 0) and the year prior (*t* = 1) were important determinants of tree growth, as has been revealed by correlational approaches (for example, Camarero and others 2011; Gazol and others 2015). However, climatic conditions further into the past (for example, *t* = 2, 3, or 4 years ago) were also important, and in many cases, rival the importance of more recent conditions (see Figure 3A–C, E, G). Among all species and sites, we generally found greater importance of past climate for silver fir. For example, temperature 4 years prior continued to affect silver fir growth at the stressed site (Figure 3A), and precipitation 3–4 years prior continued to affect silver fir growth, especially at the wet-old site (Figure 3C). Scots pine and European beech tended to show higher annual weights during the year of ring formation and the year prior for both temperature and precipitation (Figure 3D–G); although precipitation 4 years prior also continued to influence European beech growth at the wet-old site (Figure 3G).

Evaluation of the monthly importance weights reveals the main seasons or time periods during which climate is most influential to growth. The largest monthly weights for *T*^*ant*^ for silver fir occurred for the current early spring (February-April) and summer (August–September), the previous late autumn (October-December), and the summer two years prior to ring formation. Interestingly, silver fir also responded strongly to summer temperatures four years prior to ring formation at the stressed site (Figure 3A) and to growing season temperatures three years prior at the wet-young site (Figure 3B). The largest weights for *P*^*ant*^ for silver fir occurred in the late growing season and summer of the current year, the previous spring and September, and, importantly, the summers three and four years prior to ring formation (Figure 3A–C). For Scots pine and European beech, the *T*^*ant*^ and *P*^*ant*^ monthly importance weights followed a roughly similar pattern; the highest monthly weights for *T*^*ant*^ occurring mainly in the current and previous spring (April–May) and summer (August–September) (Figure 3D–G). For Scots pine at both sites and European beech at the stressed site, the largest weights for *P*^*ant*^ occurred late in the current spring-early summer (May–July) and the previous late summer (Figure 3D–G).

**Figure 4:**
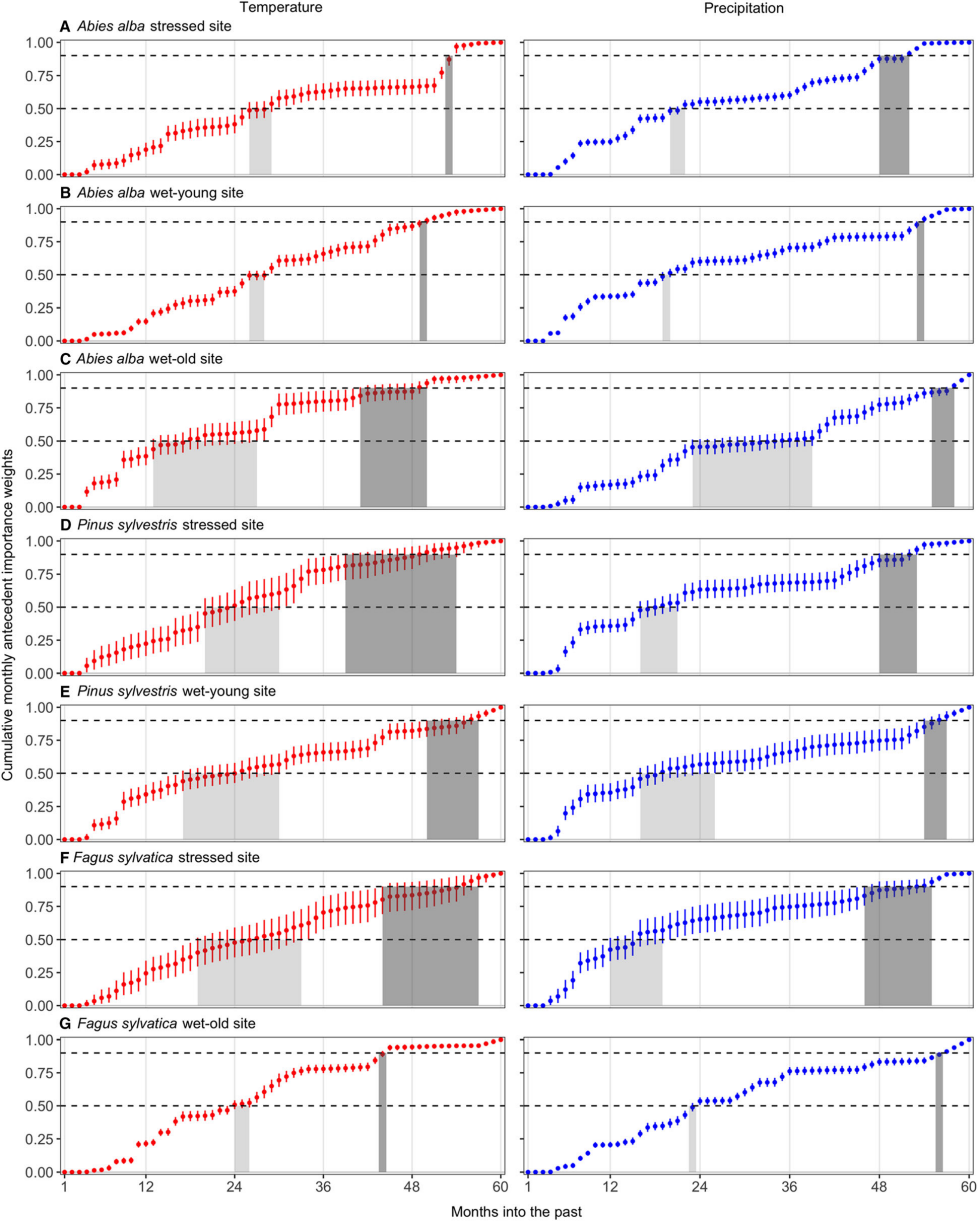
Cumulative monthly weights (posterior means and 95% Bayesian credible intervals) associated with each antecedent climate variable. Panels are organized as in Figure 3; see Figure 3 legend for definition of ‘Months into past’. The horizontal dashed lines represent the thresholds (*c* = 0.50 [lower line] and 0.90 [upper line]) used to determine the length of the memory (that is, *M*_50_ and *M*_90_, in months). The grey areas indicate the climate memory lengths (values along the “Months into past” axis) when reaching 50% (*M*_50_, light gray) or 90% (*M*_90_, dark gray) of the cumulative weights.

Based on *M*_50_ (the past time period at which the cumulative importance weights reach 50%), temperature memory (*M*_50_ = 13–26 months) was somewhat similar to precipitation memory (*M*_50_ = 12–23 months). Silver fir at the stressed and wet-young sites showed comparatively long and tightly constrained (narrow credible intervals) temperature memory (*M*_50_ = 29 months; Figure 4A, B left panel), while European beech at the wet-old site showed comparatively long and tightly constrained precipitation memory (*M*_50_ = 24 months; Figure 4G right panel). Based on *M*_90_ (cumulative importance weights reach 90%), precipitation memory was generally longer (*M*_90_ = 48–57 months) than temperature memory (*M*_90_ = 39–54 months); silver fir at the stressed site showed comparatively long and tightly constrained temperature memory (*M*_90_ = 54 months, Figure 4A left panel), and European beech at the wet-old site exhibited the long and tightly constrained precipitation memory (*M*_90_ = 57 months, Figure 4G right panel). Silver fir at the wet-old site, Scots pine, and European beech at the stressed site showed intermediate memory lengths for both *T*^*ant*^ and *P*^*ant*^ (and both *M*_50_ and *M*_90_), and their indices of memory length were less constrained, especially for *T*^*ant*^ (Figure 4).

### Tree Growth Responses to Antecedent Climate

**Figure 5:**
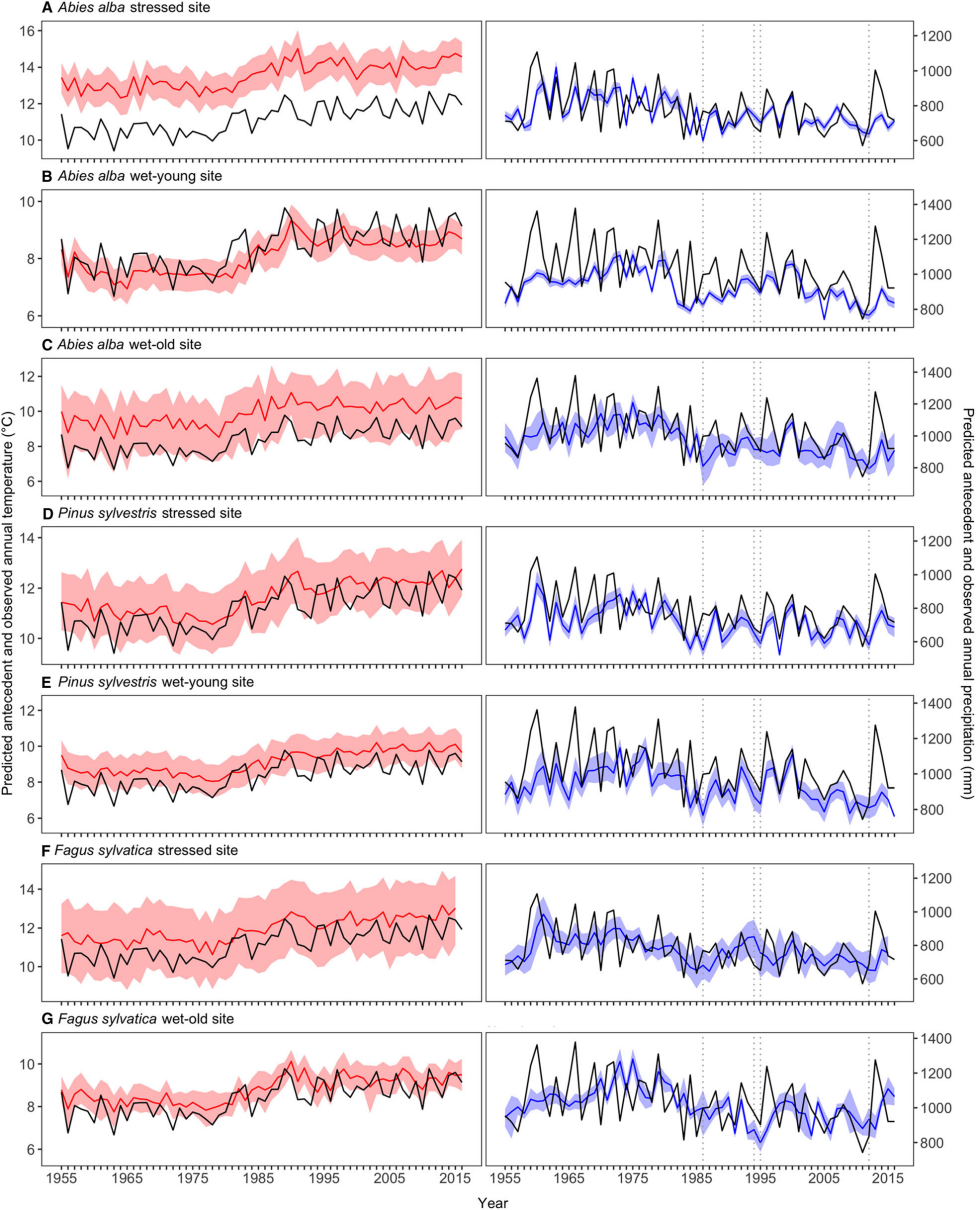
Antecedent climate variables (*T*^*ant*^ and *P*^*ant*^, with *P*^*ant*^ scaled to an annual total) governing tree growth, as predicted by the SAM model (solid colored lines are the posterior means and the shaded areas are the 95% Bayesian credible intervals), overlayed with standard summaries of the climate variables (black lines represent the annual temperature averaged across monthly means for each year [left panels] and total annual precipitation for each year [right panels]) for all species and sites. Vertical dotted lines indicate the main drought events: 1986, 1994–1995, and 2012. Note that *y*-axes scales differ among panels (species-site combinations).

Recall that the monthly importance weights describe how past monthly temperature and precipitation values are averaged to produce the antecedent climate variables that govern tree growth [see Eq. (3)]. We compared the temporal variation in these antecedent climate variables with the annual climate summaries computed from the reported monthly climate records (Figure 5). For some species-site combinations, antecedent temperature (*T*^*ant*^) was estimated to be higher than the recorded mean temperature for each year (averaged across months), indicating that tree growth was affected by warmer temperatures than registered in mean climate records (Figure 5, left column). This was particularly true for silver fir at the stressed site, where annual estimates of *T*^*ant*^ were 3 °C above the recorded annual temperatures during the study period. A similar but weaker pattern was found for silver fir at the wet-old site and Scots pine and European beech. In contrast, *T*^*ant*^ was slightly lower than the recorded mean temperature for several years for silver fir trees growing at the wet-young site, meaning that not only warm summer months but also cooler months were key for tree growth, depending upon the site.

Antecedent precipitation (*P*^*ant*^) followed a similar trend among all species and sites, with trees responding to slightly lower values than the recorded annual precipitation totals during the study period (Figure 5, right column). Based on annual precipitation data, 1986, 1994–1995, and 2012 represent years with comparatively lower precipitation amounts and reduced growth rates; interestingly, the predicted *P*^*ant*^ values were lower than the recorded totals before, during, and after these dry years for most sites and species (see for instance *P*^*ant*^ values in 1986 for silver fir and Scots pine at the stressed site and *P*^*ant*^ values in 1994 for European beech at the wet-old site), indicating that the months during which precipitation has the greatest influence on tree growth were particularly dry.

## Discussion

### Precipitation and Temperature Legacy Effects on Tree Growth

Related to our first and third research questions (Q1 and Q3), we found that antecedent precipitation (*P*^*ant*^) and temperature (*T*^*ant*^) were significant predictors of tree radial growth across all study species and sites (Figure 2C, D), and often interacted with each other (Figure 2E) and/or with harvesting intensity (*HI*) (Figure 2H, I) to influence tree growth. Across all species, warmer conditions (higher *T*^*ant*^) generally led to greater growth under average moisture and forest harvesting. This overall positive effect of *T*^*ant*^ is generally expected given that *T*^*ant*^ represents a long-term, integrated index of growth temperature, and physiological and enzymatic processes underlying carbon acquisition, allocation, and biomass production are generally stimulated by warmer temperatures via enhancement of metabolic rates and/or extending the growing season (Tardif and others 2003; Michelot and others 2012; Galván and others 2014; Camarero and others 2015b; González de Andrés and others 2015). In most cases, tree growth was also stimulated by higher precipitation under average temperature and harvesting (positive effect of *P*^ant^), which is also consistent with other studies that show a positive relationship between tree-ring widths and precipitation (Aussenac 2002; Peguero-Pina and others 2007; Tegel and others 2014; Gazol and others 2015).

However, tree growth responses to antecedent climate are a bit more complex given that other climate factors and past harvesting practices may govern responses to a particular antecedent climate variable (for example, see the net sensitivities in Appendix S9). For example, while growth of most species at most sites is simulated by higher antecedent precipitation, growth of European beech at the wet-old site is predicted to be reduced by higher precipitation under certain conditions, such as under high levels of harvesting and during warmer than average periods. Why would this occur? The negative effect of precipitation on growth may reflect an indirect effect of cloudy conditions at these wet and cool sites (Rozas and others 2015). Wetter and warmer conditions could also shift carbon allocation towards other processes such as reproduction and masting (Hacket-Pain and others 2018), which could lead to reducing radial growth. Additionally, warmer antecedent conditions tend to stimulate growth in silver fir at the wet-young site, except during dry periods associated with little/no forest harvesting, in which case, any increase in temperature is expected to reduce growth. Under such conditions, competition for water should be high (assuming stand density may be relatively high in the absence of forest harvesting), and increasing temperatures would likely exacerbate drought-type conditions (Vicente-Serrano and others 2014), thus reducing tree growth (Jump and others 2017).

### Timescales of Influence of Climate on Tree Growth

Interpretation of the effects of climate (for example, precipitation and temperature) on tree growth also requires consideration of the timescales over which these drivers influence growth, thus further addressing Q1 and associated hypothesis H1. Our analysis indicated that temperature and precipitation conditions as far back as 2–4 years prior to ring formation continue to influence ring widths (Figures 3 and 4). Our ecologically focused sampling design may explain these long time-scales of influence, which were comparatively long for silver fir. Other studies implementing the SAM model with tree-ring width data found shorter climate memory (Peltier and others 2018), potentially due to the use of tree-ring data from a more traditional dendrochronological sampling approach that primarily focused on precipitation reconstruction, which may introduce biases in the quantification of growth responses to environmental variation (Nehrbass-Ahles and others 2014; Klesse and others 2018). In addition, the longer climatic memory uncovered in this study is consistent with prior findings that climate as far back as six years was a robust predictor of silver fir growth (Becker 1989).

Why such long climatic memory in species such as silver fir? The underlying physiological mechanisms may be partly related to needle retention times, which can be up to 10 years for silver fir under both favorable growing and suppressed conditions (Withington and others 2006). Favorable years may stimulate greater production of needles, and likewise, climatic stresses may trigger needle shedding or reduce needle production, resulting in an increase (favorable years) or decrease (stressful years) in available photosynthates (Robakowski and Bielinis 2017), in-turn affecting growth (Fritts 1976) for multiple years after such events occur. Further, old needles may be a reservoir of stored leaf nitrogen, and loss of such needles could reduce nitrogen availability in subsequent needle cohorts (Balster and Marshall 2000; Wyka and others 2016), which would likely have an effect on radial growth as well. Interestingly, precipitation three to four years prior to ring formation was estimated to be more important than precipitation received two years prior for silver fir (Figure 3a–c). There are several potential explanations to interpret these changes in the antecedent importance weights for the different years. The total importance weight for the fall/winter period (that is, Oct.–Feb./March) is relatively high for all years for silver fir, indicating that winter/fall precipitation received over the past four years is important to growth in this species. It is possible that winter precipitation received during different past time periods influenced growth through different mechanisms that are not explicitly described by the model. The importance of winter precipitation received further in the past could be linked to deep soil water recharge dynamics and silver fir’s rooting behavior, which is expected to develop comparatively deep roots to access water in deeper layers under warm-dry conditions (Lebourgeois and others 2013; Grossiord and others 2014; Gazol and Camarero 2016; Brinkmann and others 2019). Trees are relatively inactive and do not use much water during the winter, so water delivered by winter precipitation events can infiltrate deeper and be accessed later (for example, when surface soils are dry), rather than being immediately lost via transpiration. On the other hand, more recent precipitation may directly affect growth by influencing available soil moisture, which in-turn affects photosynthesis and carbohydrate production and allocation. Precipitation received further back in time (for example, 3–4 years ago) may also have had a greater effect on morphological or structural properties of the tree, such as xylem conduit size and biomass of needles produced, which can continue to influence growth for many years (Pellizzari and others 2016; Sass and Eckstein 1995). Furthermore, silver fir is particularly vulnerable to drought-triggered dieback (Gazol and others 2015), and further research could investigate if this vulnerability is linked to its long climatic memory or its reliance on winter precipitation received over multiple years.

Compared to the other species, Scots pine generally showed shorter climatic memory, in line with other pine species (Peltier and others 2018). In particular, Scots pine appears particularly sensitive to moisture conditions associated with the summer months during the year of ring formation (see the high antecedent importance weights estimated for this period, Figure 3D, E, right panels). This comparatively short memory agrees with reports that warmer and wetter conditions during the year of ring formation stimulate Scots pine growth (Tardif and others 2003; Sánchez-Salguero and others 2015), and drought constrains the physiological activity of Scots pine during this period (Eilmann and Rigling 2012; Gea-Izquierdo and others 2014; Lévesque and others 2014). This evergreen, shade-intolerant species has strong stomatal control of transpirational water loss under dry conditions (Zweifel and others 2009), with a higher drought tolerance than the other two species. Because growth is tied to physiological behavior and carbohydrate storage and allocation (Hoch and Körner 2003; Ogle and Pacala 2009), we would expect ring widths to also be strongly influenced by the environmental conditions experienced during the year of ring formation.

We also detected comparatively shorter climatic memory in European beech at the stressed site, in agreement with Anderegg and others (2015) and Camarero and others (2018), both of which found shorter legacies in angiosperms than in gymnosperms. Similar to European beech in Mediterranean dry sites (Tegel and others 2014; Hacket-Pain and others 2016), growth in the stressed site was stimulated by current May and summer precipitation. However, growth of European beach at the wet-old site showed longer and more constrained precipitation legacies (Figure 4G, right panel), and its growth was reduced by spring precipitation, similar to observations from relatively cold sites (Dittmar and others 2003; Rita and others 2014). Climate conditions during the year prior to ring formation were also important for European beech growth at this site, which agrees with other reports that growth reductions in this species are linked to climate conditions in the prior year (Serra-Maluquer and others 2019), partly due to a shift in carbon allocation towards reproduction (mast years) (Hacket-Pain and others 2017; Müller-Haubold and others 2013).

### Mismatch Between Influential Climate and Observed Climate

While forests are experiencing rising temperatures worldwide (Kirilenko and Sedjo 2007; IPCC 2014), forest productivity may be most strongly influenced by extremes in seasonal temperatures or moisture stresses (Smith 2011). That is, it is unlikely that estimates of average temperature or precipitation conditions are indicative of the actual conditions driving tree growth. The SAM framework revealed particular time periods (specific past months or seasons of a 5-year period) that are most influential to tree growth, via the antecedent importance weights, which in turn determine the antecedent climate covariates [for example, *T*^*ant*^ and *P*^*ant*^, Eq. (3)]. Related to our second research question (Q2) and hypothesis (H2), we evaluated the temporal trends in these antecedent climate variables produced by the SAM model and compared them to mean climate records. For example, because of the unequal contribution of temperature during different time periods (Figure 3), the *T*^*ant*^ conditions influencing tree growth were warmer than the yearly estimates of mean temperature, for nearly all years of the study period (Figure 5, left column). This mismatch is especially pronounced for silver fir at the stressed site (Figure 5A), reflecting the dominant influence of temperature during warmer months or seasons (see monthly *T*^*ant*^ weights, Figure 3). The highest temperature weights tended to occur in the spring or summer of the current and previous year, suggesting temperature influences annual tree-ring widths partly through modifying growing season length, via early and warm springs, or increasing late-season drought stress (Peltier and Ogle 2019a; Vitali and others 2018).

There also appears to be a general trend towards increasing drought stress during the critical time periods during which precipitation is most influential to tree growth. For example, the annual estimates of antecedent precipitation (*P*^*ant*^) generally overlap with the total annual precipitation received each year during the earlier part of the study period (prior to 1985–1990), but after this period, *P*^*ant*^ is often lower than the reported precipitation totals (Figure 5, right column). In fact, rising temperatures and severe drought events characterize the recent, post-1985 period in the Spanish Pyrenees (Camarero and others 2011; Carnicer and others 2019), which have triggered dieback in some lower elevation populations of silver fir (Sangüesa-Barreda and others 2015; Gazol and Camarero 2016). Interestingly, the *P*^*ant*^ values were particularly low before, during, and after 1986, 1994–1995, and 2012 drought events, for most populations. Given the timescales of influence of precipitation (Figures 2 and 3), this suggests that the impact of these drought events persisted for multiple years given the comparatively long duration of their influence, pointing to notable drought legacy effects. Additionally, the year-to-year variability in *P*^*ant*^ was lower than the year-to-year variability in the reported total annual precipitation, reflecting the buffering capacity of trees such that their climatic memory generally results in a reduced impact of extreme wet or dry years.

### Historical Management Modifies Tree Growth and Its Response to Climate

Regarding our third research question (Q3) and hypothesis (H3), past management in these forests modified the responses of tree growth to climate, but these effects differed across species and sites (see first section in Discussion). Under continued climate change, management practices such as forest harvesting may be employed to help regulate species and individuals’ competitive interactions.

Intensive harvesting reduced silver fir growth, probably as a result of past practices that removed larger trees, leaving behind less vigorous trees with reduced capacity to adjust to the warmer and drier conditions of the late twentieth century (Oliva and Colinas 2007). Management practices particularly affected silver fir trees at the stressed site, where harvesting intensity was fairly high due to higher wood quality and easier extraction (Cabrera 2001). The negative effect of intense management in the silver fir populations of this region has been associated with several drought events in 1986 and subsequent years, which led to increased mortality and canopy dieback (Camarero and others 2011; Sangüesa-Barreda and others 2015). Nevertheless, harvesting promoted young silver fir tree growth under wet and warm conditions, as also suggested by the significant positive *HI* × *P*^*ant*^ and *HI* × *T*^*ant*^ interactions; such harvesting likely enhanced growth in this population by reducing competition for soil moisture and light, allowing younger trees to expand their crowns and fill gaps created by harvesting (Bottero and others 2017).

Management treatments also helped to mitigate the impacts of extreme droughts on Scots pine. Scots pine growth was enhanced by past harvesting, particularly under cooler conditions, suggesting these trees were responding primarily to release from competition, as long as sufficient moisture was available. Scots pine growth is usually reduced in denser stands, where competitive effects are expected to be stronger (García-Abril and others 2007; Sohn and others 2016; Del Río and others 2017). These findings agree with previous studies reporting high growth rates and greater climatic sensitivity in managed plots (Mäkinen and Isomäki 2004; Primicia and others 2016).

European beech growth tended to decrease with harvesting intensity at the stressed site, in opposition to expected positive effects of competitive release. Because beech requires humid conditions, reducing tree cover could have enhanced soil evaporation rates, leading to greater drought stress and decreased growth at the stressed site (Joffre and Rambal 1993). However, harvesting might favor European beech growth at the wet-old site, especially under drier and cooler conditions, as indicated by the negative *HI* × *P*^*ant*^ and *HI* × *T*^*ant*^ effects. The positive effect of harvesting under comparatively dry conditions at these wetter sites could, again, reflect reduced competition with greater harvesting (Gessler and others 2007). Likewise, the positive effect of harvesting on growth during cooler periods could reflect the effect of sparser stands whereby greater interception of solar radiation by the forest floor and canopy may have led to warmer microenvironments that could have stimulated greater growth (Dittmar and others 2006).

### Limitations, Caveats, and Further Research

Ideally, we would also have treated management data similar to our treatment of the climate data, explicitly estimating forest management memory via the stochastic antecedent weights. However, the temporal resolution of the historical forest records made this impossible. Though we did implement the same analysis defining harvesting intensity as the percentage of volume harvested, the model for European beech at the wet-old site proved to be computationally infeasible (results for other species and sites are summarized in Appendix S10). Nevertheless, our model represents one of the first attempts to understand how climatic memory and management history interact to govern tree growth responses, across multiple species and sites, over a period of more than 50 years. Thus, historical data, such as the forest management records used in this study, combined with tree-ring data present a unique opportunity to reconstruct the factors affecting forest productivity. Although our SAM models estimated the antecedent effects for temperature, precipitation, and their interaction, future work could include other climatic variables based on more physiologically motivated indices of water availability and drought stress.

## Conclusions

Our results highlight the importance of evaluating the ecological memory associated with climate and historical management for disentangling differential responses of tree productivity to environmental changes. We uncovered multi-year climate legacies affecting tree growth, and besides these legacy effects were modulated by historical forest management practices. Among the three species considered, silver fir and European beech exhibited the longest climatic memory and the strongest interaction between management and climate legacies. Our results also identify critical time periods controlling tree growth and suggest that climatic memory can help buffer trees against large variability in climatic conditions, but can also extend the impact of specific climatic events. Our findings improve understanding of species-specific differences in climatic responses that can underlie mechanisms of coexistence in a fluctuating climate (Kelly and Bowler 2002), pointing to the need for management strategies adapted to species-specific responses and site conditions. Although many researchers have repurposed dendrochronology datasets to understand tree growth-climate relationships, our work with tree cores from an ecological sampling design shows that climatic memory can be much longer than commonly assumed. Our study emphasizes the importance of accounting for past climate and forest management when modelling tree growth and productivity (Kolus and others 2019).

## Supplementary Information

Below is the link to the electronic supplementary material.

Supplementary file 1 (PDF 1614 KB)
