# Supplementary material for: Disentangling the Legacies of Climate and Management on Tree Growth
Source: Ecosystems. 2021 Jun 22;25(1):215–35. doi: 10.1007/s10021-021-00650-8 (PMC8827397; doi:10.1007/s10021-021-00650-8)

Supplementary Information

Disentangling the legacies of climate and management on tree growth

Laura Marqués, Drew M. P. Peltier, J. Julio Camarero, Miguel A. Zavala, Jaime Madrigal-González, Gabriel Sangüesa-Barreda & Kiona Ogle

Appendix S1. Soil properties in the three study sites.

Site	Sands (%)	Silt (%)	Clays (%)	Soil pH
Paco Ezpela (PE) (Stressed site)	45.58	41.20	13.22	6.0
Las Eras (LE) / Gamueta (GA) (Wet sites)	27.21	56.44	16.35	6.5

Appendix S2. Time series plots of average tree-ring widths for the three tree species (*Abies alba*, *Pinus sylvestris* and *Fagus sylvatica*) in the study sites (stressed, wet-young and wet-old sites).

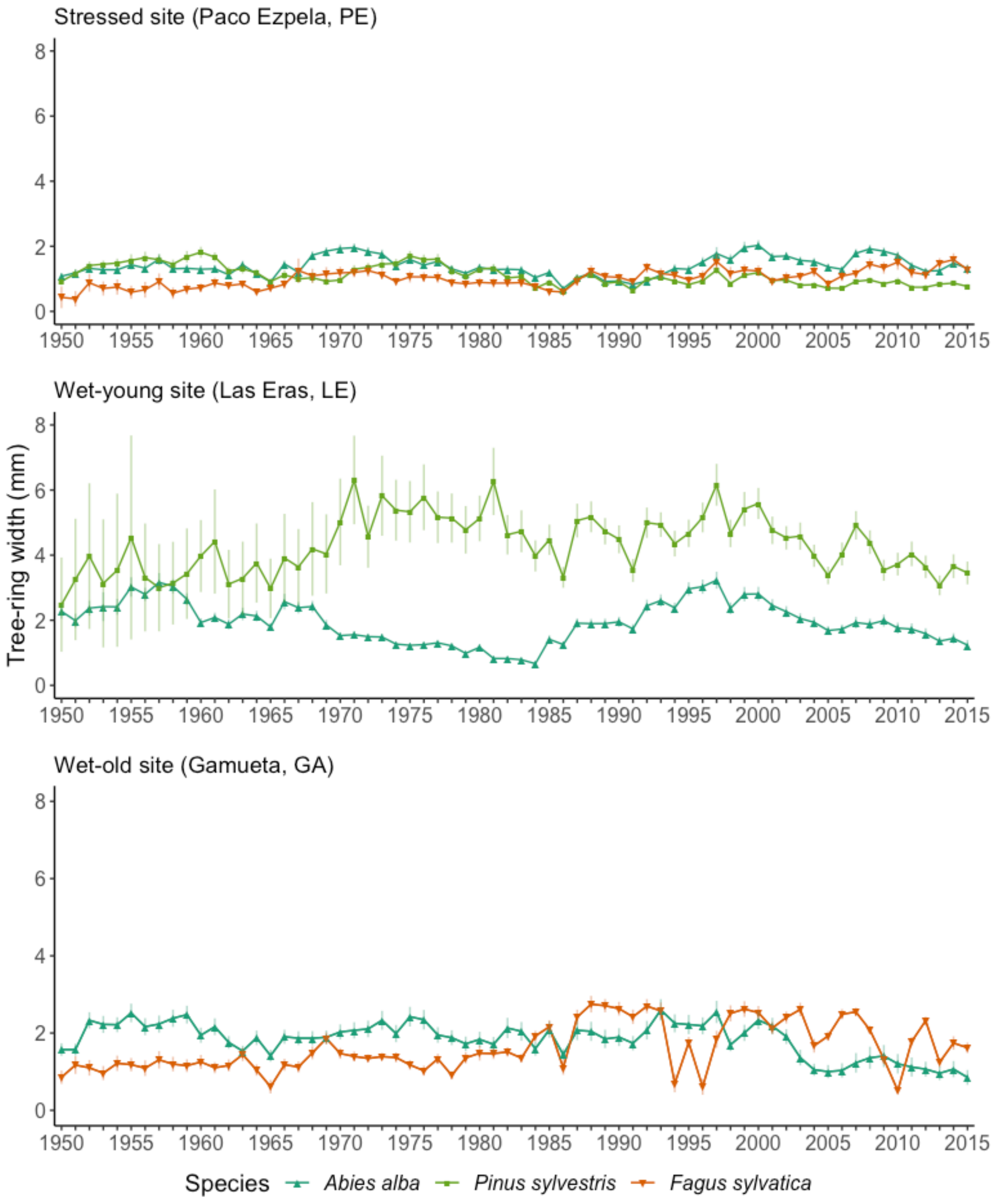

Appendix S3. Cross-dating statistics for the tree-ring series.

Site	Tree species	Correlation with master series	Correlation between trees	Best-replicated period	Expressed Population Signal	Variance in first eigenvector (%)
Paco Ezpela	*Abies alba*	0.59	0.35	1944-2016	0.95	37.2
	Pinus sylvestris	0.47	0.27	1942-2016	0.86	27.4
	Fagus sylvatica	0.42	0.25	1967-2016	0.87	29.4
Las Eras	*Abies alba*	0.63	0.50	1952-2016	0.98	64.7
	Pinus sylvestris	0.52	0.26	1978-2016	0.85	38.1
Gamueta	*Abies alba*	0.49	0.30	1863-2016	0.88	36.8
	Fagus sylvatica	0.63	0.49	1961-2016	0.96	52.4

The Expressed Population Signal (EPS) measures how well replicated is a chronology or mean site series. A threshold value of EPS > 0.85 is usually considered to define well-replicated chronologies (Wigley et al. 1984).

Wigley TML, Briffa KR, Jones PD (1984) On the average value of correlated time series, with applications in dendroclimatology and hydrometeorology. J Appl Meteorol 23:201-213.

Appendix S4. Stand density, stand volume, harvested trees, and harvested volume for each target species at each study site. Colors correspond to different species and columns to different sites.

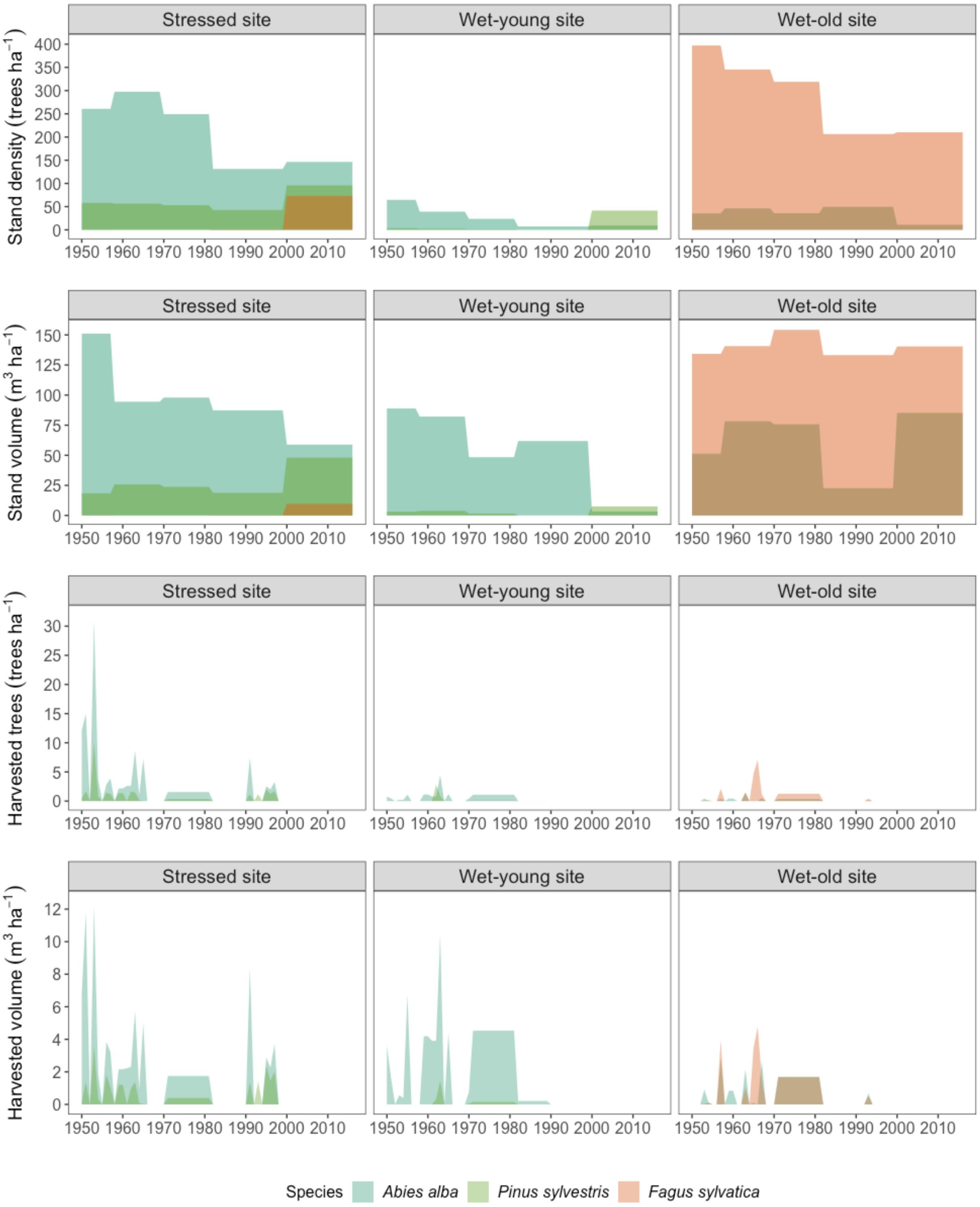

Appendix S5. SAM model code implemented in JAGS.

R Packages

```
library(dplR)
library(vegan)
library(rjags)
load.module("dic")
library(ggplot2)
library(mcmcplots)
library(coda)
```

1.- Construct antecedent variables

```

model{
for(v in 1:Nv){ # variable 1=precipitation, 2=temperature
    for(t in 1:Nlag){ # lag (in years) up to 5
    for(m in 1:12){ # month 1-12
        ant.Mean1[m,t,v] <- delta[m,t,v]*((v==1)*meanppt[m]+(v==2)*meantave[m])
        ant.Mean1.real[m,t,v] <- weight[m,t,v]*((v==1)*meanppt[m]+(v==2)*meantave[m])
      }
      ant.Mean2[t,v]<-sum(ant.Mean1[1:12,t,v])
      ant.Mean2.real[t,v]<-sum(ant.Mean1.real[1:12,t,v])
    }
    ant.Mean[v]<-sum(ant.Mean2[1:5,v])
    ant.Mean.real[v]<-sum(ant.Mean2.real[1:5,v])
}
# Compute antecedent climate variables for time "block" into the past t
  for(v in 1:Nv){
    for(j in 1:Nblocks){ #block, 1-38
      deltaX[j,v] ~ dgamma(1,1)
      weightX[j,v] <- deltaX[j,v]/sum(deltaX[,v])
    }
  }
# Compute sum of deltas needed for computing importance weights
  for(v in 1:Nv){
    for(t in 1:Nlag){
      sumD1[t,v] <- sum(delta[,t,v]) # sum deltas across months
    }
    sumD[v] <- sum(sumD1[,v]) # sum deltas across lags
  }
# Compute importance weights of interest
  for(v in 1:Nv){
    for(t in 1:Nlag){
      # Compute yearly weights
      yr.w[t,v] <- sum(weight[,t,v])
      # Define monthly importance weights for every month x year into past:
     for(m in 1:12){
     delta[m,t,v] <- (deltaX[block[t,m],v]/BlockSize[t])*(1-(t==1)*step(m-9.5))
     weight[m,t,v] <- delta[m,t,v]/sumD[v]
     weightOrdered[(t-1)*12 + (12-m+1),v] <- weight[m,t,v]
      }
    }
  }

# Construct the antecedent variable
# We use a computational "trick" to speed convergence time, multiplying the climate data by
the nonidentifiable weight (delta) rather than weight.
```

```
for(v in 1:Nv){
    for(t in 1:Nlag){
      for(m in 1:12){
        for(i in 1:Nyears){
          antX1[i,m,t,v] <- delta[m,t,v]*((v==1)*ppt[(i+5)-t+1, 1+m] +
                                          (v==2)*tave[(i+5)-t+1, 1+m])
# Compute the real antX
          antX1.real[i,m,t,v] <- weight[m,t,v]*((v==1)*ppt[(i+5)-t+1, 1+m] +
                                                (v==2)*tave[(i+5)-t+1, 1+m])
        }
      }
    }
  }
# Compute the antecedent variable by summing the weighted climate variables
  for(v in 1:Nv){
    for(i in 1:Nyears){
      for(t in 1:Nlag){
        ant.sum1[i,t,v] <- sum(antX1[i,,t,v])
        ant.sum1.real[i,t,v] <- sum(antX1.real[i,,t,v])
      }
      # Monitore the ant.sum2.real to extract antX changes over time
      ant.sum2[i,v] <- sum(ant.sum1[i,,v])
      ant.sum2.real[i,v] <- sum(ant.sum1.real[i,,v])
      antX[i,v] <- ant.sum2[i,v] - ant.Mean[v]
      antX.real[i,v] <- ant.sum2.real[i,v] - ant.Mean.real[v]
    }
  }
# Compute cumulative monthly weights
  for(v in 1:Nv){
    for(t in 1:(12*Nlag)){
      cum.weight[t,v] <- sum(weightOrdered[1:t,v])
    }
    for(m in 1:12){
      for(t in 1:Nlag){
        alpha[m,t,v] <- weight[m,t,v]/yr.w[t,v]
      }
    }
  }
```

2.- Likelihood for log tree-ring width data:

```
for(i in 1:Nobs){
    LogWidth[i] ~ dnorm(mu.LogWidth[i], tau)
    LogWidth.rep[i] ~ dnorm(mu.LogWidth[i], tau) # Replicated data for evaluating model fit
    sq.diff[i]<-pow(LogWidth.rep[i]-LogWidth[i],2)
    resid[i]<-LogWidth[i]-mu.LogWidth[i]
    Dsum<-sum(sq.diff[]) #Total sum of squared differences
```

3.- Mean model with effect of age, antecedent climate effects (antX) and past ring-width (LogAR1)

```
mu.LogWidth[i] <- a[1,CoreID[i]] + a[2,CoreID[i]]*Age[i] + a[3,CoreID[i]]*antX[yrID[i],1] +
a[4,CoreID[i]]*antX[yrID[i],2] + a[5,CoreID[i]]*antX[yrID[i],1]*antX[yrID[i],2] +
a[6,CoreID[i]]*(LogAR1[i]-log(Meanwidth+1)) + a[7,CoreID[i]]*HI[i] +
a[8,CoreID[i]]*HI[i]*antX[yrID[i],1] + a[9,CoreID[i]]*HI[i]*antX[yrID[i],2]
LogAR1[i] ~ dnorm(0,0.0001) # For missing data
}
```

4.- Compute identifiable parameters

```
  for(c in 1:Ncores){
    a_star[1,c]<-a[1,c]
    a_star[2,c]<-a[2,c]
    a_star[3,c]<-a[3,c]*sumD[1]
    a_star[4,c]<-a[4,c]*sumD[2]
    a_star[5,c]<-a[5,c]*sumD[1]*sumD[2]
    a_star[6,c]<-a[6,c]
    a_star[7,c]<-a[7,c]
    a_star[8,c]<-a[8,c]*sumD[1]
    a_star[9,c]<-a[9,c]*sumD[2]
  }
   mu.a_star[1]<-mu.a[1]
   mu.a_star[2]<-mu.a[2]
   mu.a_star[3]<-mu.a[3]*sumD[1]
   mu.a_star[4]<-mu.a[4]*sumD[2]
   mu.a_star[5]<-mu.a[5]*sumD[1]*sumD[2]
   mu.a_star[6]<-mu.a[6]
   mu.a_star[7]<-mu.a[7]
   mu.a_star[8]<-mu.a[8]*sumD[1]
   mu.a_star[9]<-mu.a[9]*sumD[2]
```

5.- Prior distributions

Assign hierarhical priors to the core-level effects in the mean model, and assign relatively non-informative priors to population-level parameters

```
  # Intercept
  for(c in 1:Ncores){
    # Core-level hierarchical prior
    a[1,c] ~ dnorm(mu.a[1],tau.a[1])T(0,4)
  }
  # Age effect
  for(c in 1:Ncores){
    # Core-level hierarchical prior
    a[2,c] ~ dnorm(mu.a[2],tau.a[2])T(,0)
  }
  # Other parameters
  for(k in 3:9){
    for(c in 1:Ncores){
      # Core-level hierarchical prior
      a[k,c] ~ dnorm(mu.a[k],tau.a[k])
    }
  }
  # Sigs
  for(k in 1:9){
    tau.a[k] <- pow(sig.a[k],-2)
    sig.a[k] ~ dunif(0,100)
  }
  # Hierarchical means
  mu.a[1] ~ dnorm(0,0.0001)T(0,4)
  mu.a[2] ~ dnorm(0,0.0001)T(,0)
  for(k in 3:9){
    mu.a[k] ~ dnorm(0,0.0001)
  }
  sig ~ dunif(0,100)
  tau <- pow(sig,-2)
}
```

Appendix S6. Trace plots for assessing convergence for the population parameters μ_{α_k}. Convergence was formally assessed via the potential scale reduction factor (Gelman and Rubin 1992), for all parameters, not just the population-level parameters (see methods section).

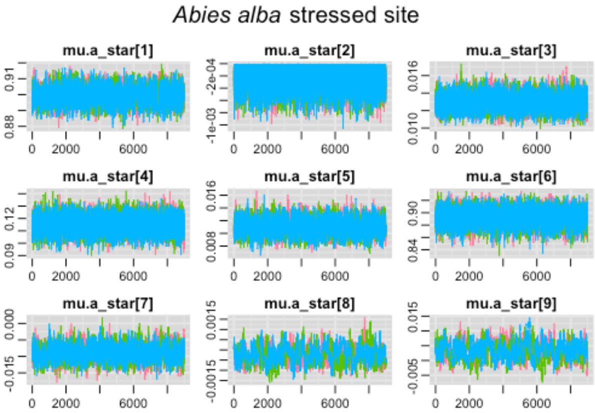
Abies alba stressed site
mu.a_star[1]
mu.a_star[2]
mu.a_star[3]
mu.a_star[4]
mu.a_star[5]
mu.a_star[6]
mu.a_star[7]
mu.a_star[8]
mu.a_star[9]

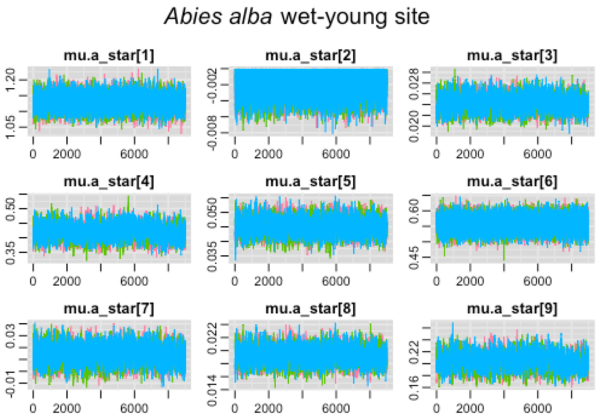
Abies alba wet-young site
mu.a_star[1]
mu.a_star[2]
mu.a_star[3]
mu.a_star[4]
mu.a_star[5]
mu.a_star[6]
mu.a_star[7]
mu.a_star[8]
mu.a_star[9]

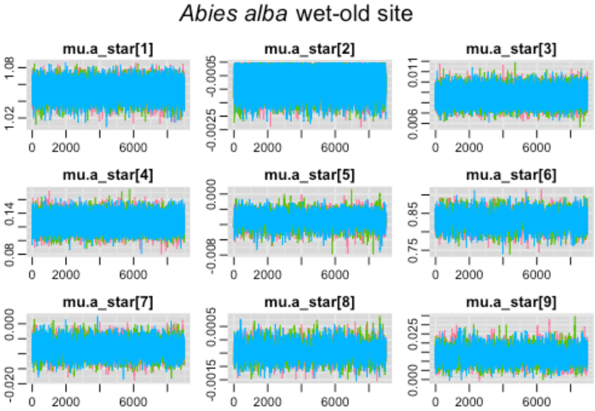
Abies alba wet-old site
mu.a_star[1]
mu.a_star[2]
mu.a_star[3]
mu.a_star[4]
mu.a_star[5]
mu.a_star[6]
mu.a_star[7]
mu.a_star[8]
mu.a_star[9]

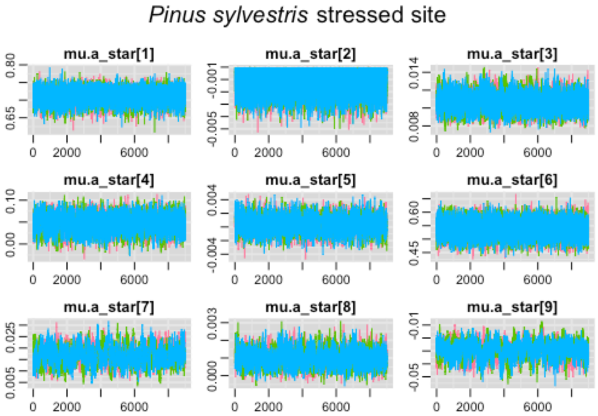
Pinus sylvestris stressed site
mu.a_star[1]
mu.a_star[2]
mu.a_star[3]
mu.a_star[4]
mu.a_star[5]
mu.a_star[6]
mu.a_star[7]
mu.a_star[8]
mu.a_star[9]

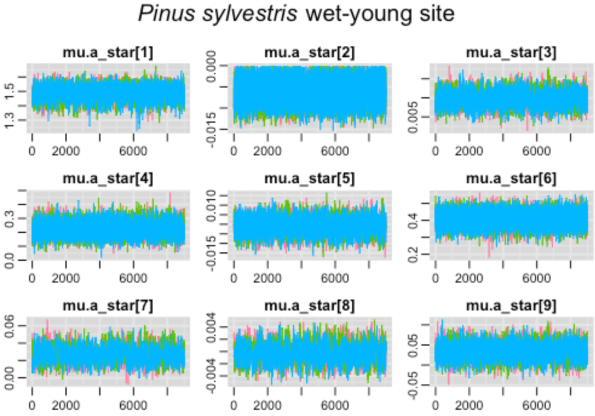
Pinus sylvestris wet-young site
mu.a_star[1]
mu.a_star[2]
mu.a_star[3]
mu.a_star[4]
mu.a_star[5]
mu.a_star[6]
mu.a_star[7]
mu.a_star[8]
mu.a_star[9]

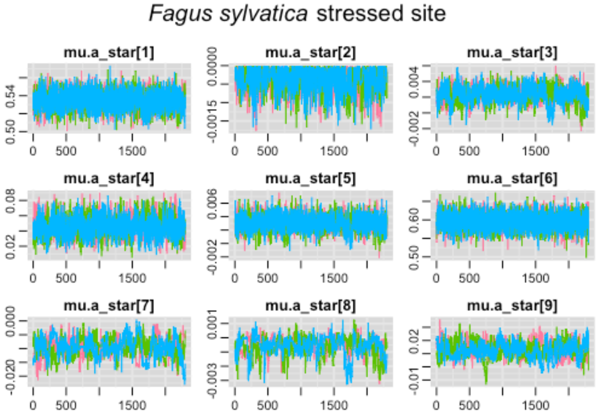
Fagus sylvatica stressed site
mu.a_star[1]
mu.a_star[2]
mu.a_star[3]
mu.a_star[4]
mu.a_star[5]
mu.a_star[6]
mu.a_star[7]
mu.a_star[8]
mu.a_star[9]

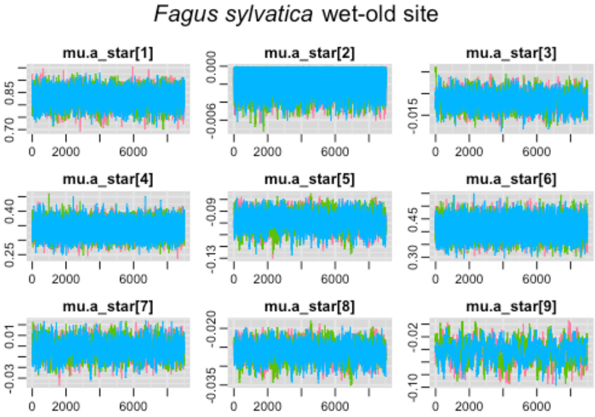
Fagus sylvatica wet-old site
mu.a_star[1]
mu.a_star[2]
mu.a_star[3]
mu.a_star[4]
mu.a_star[5]
mu.a_star[6]
mu.a_star[7]
mu.a_star[8]
mu.a_star[9]

Appendix S7. Plots of observed vs. predicted (replicated data) tree growth (*G*) for each species-site combination. Coefficients of determination (R^2) from regressions of observed vs. predicted growth values, defined as $G = \log(r+1)$, are shown above each panel.

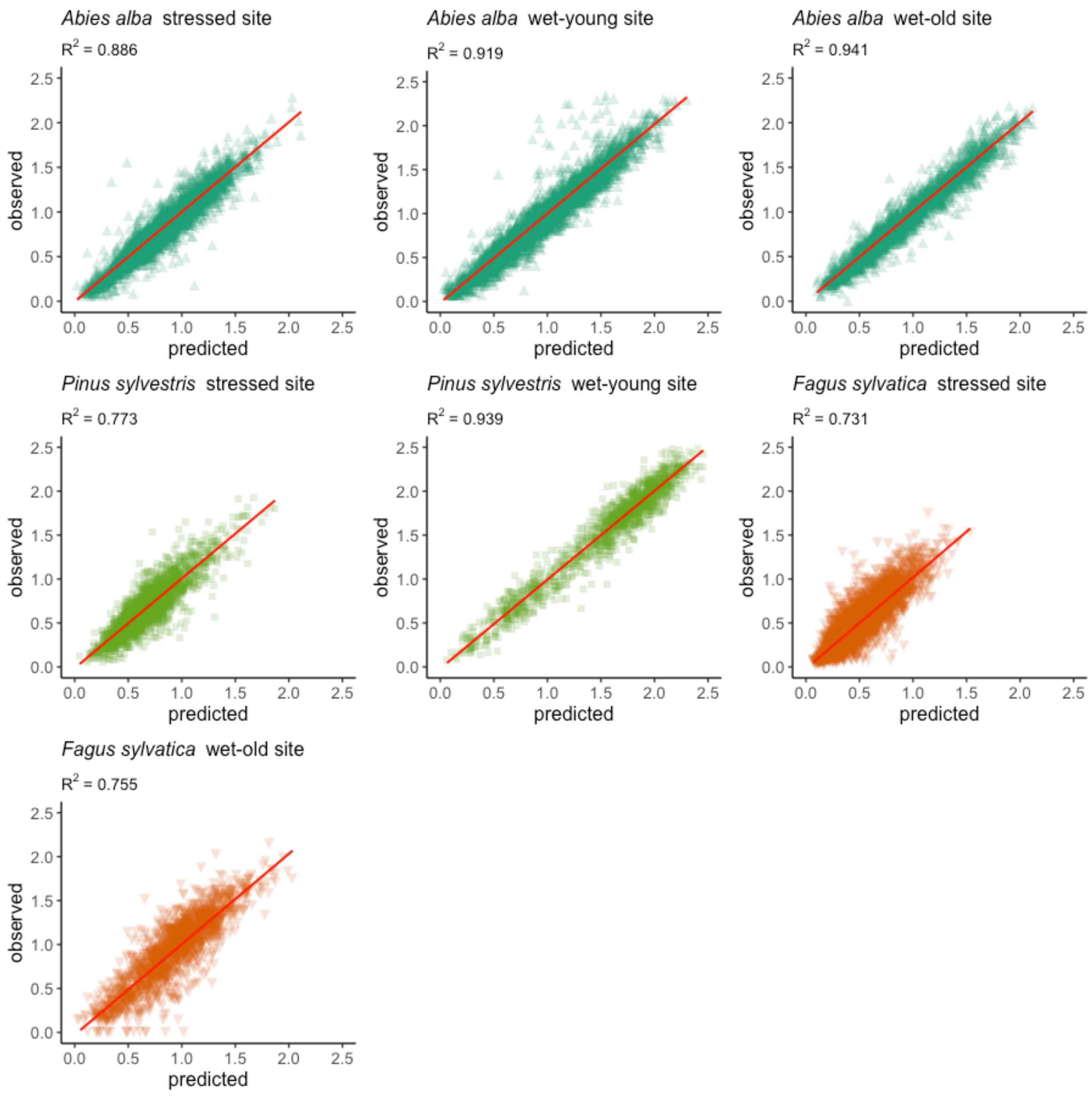

Appendix S8. Relationships between mean tree-ring width and tree age for each species-site combination over the period 1950-2016 represented up to 100 years.

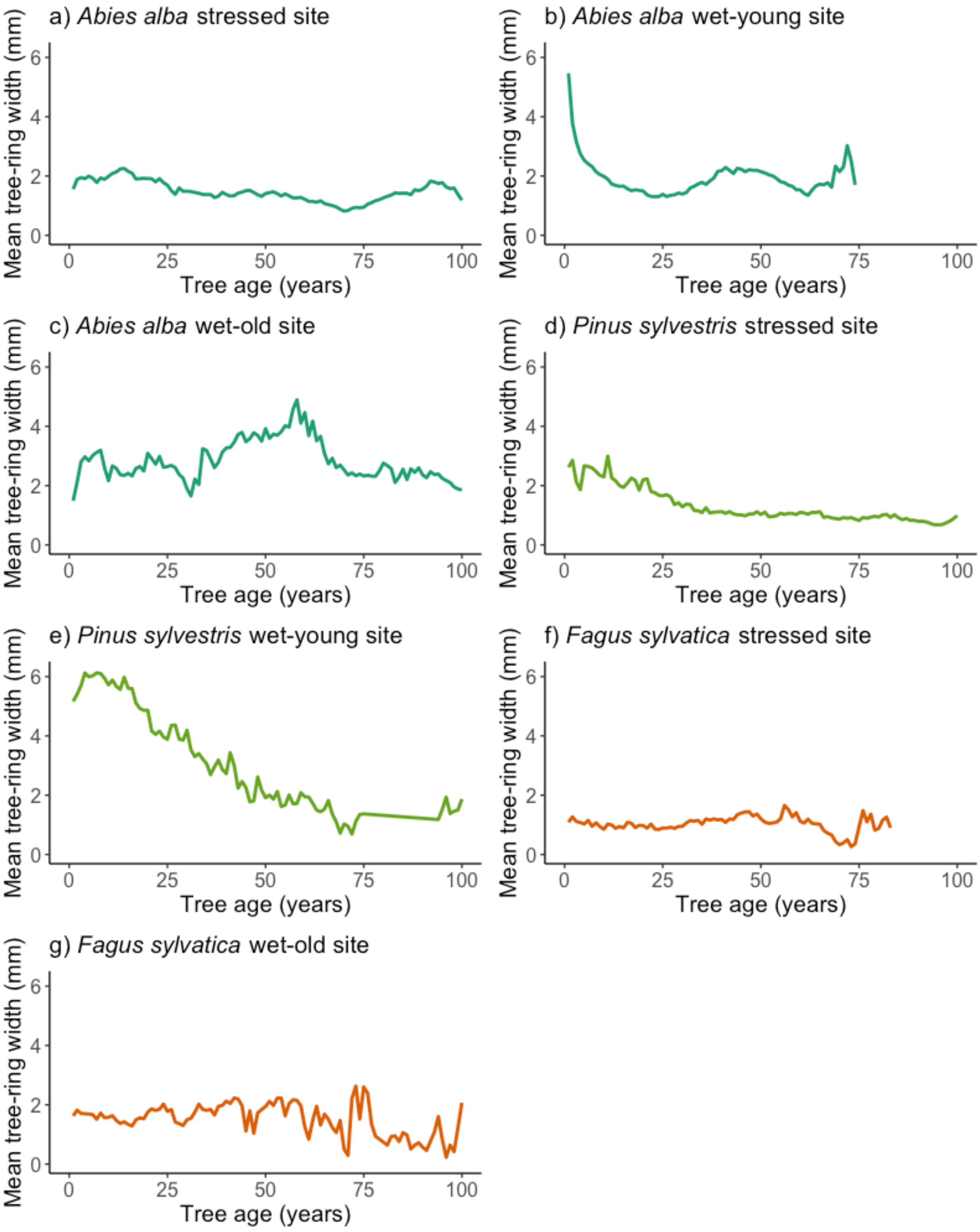

Appendix S9. Estimates of the net sensitivity of tree growth (G, see Eqn 1) to changes in P^{ant} (a, b) and T^{ant} (c, d), under minimum (a, c) and maximum (b, d) values of harvesting intensity in terms of number of trees removed from the stand (*HI*) for each target tree species in the three study sites.

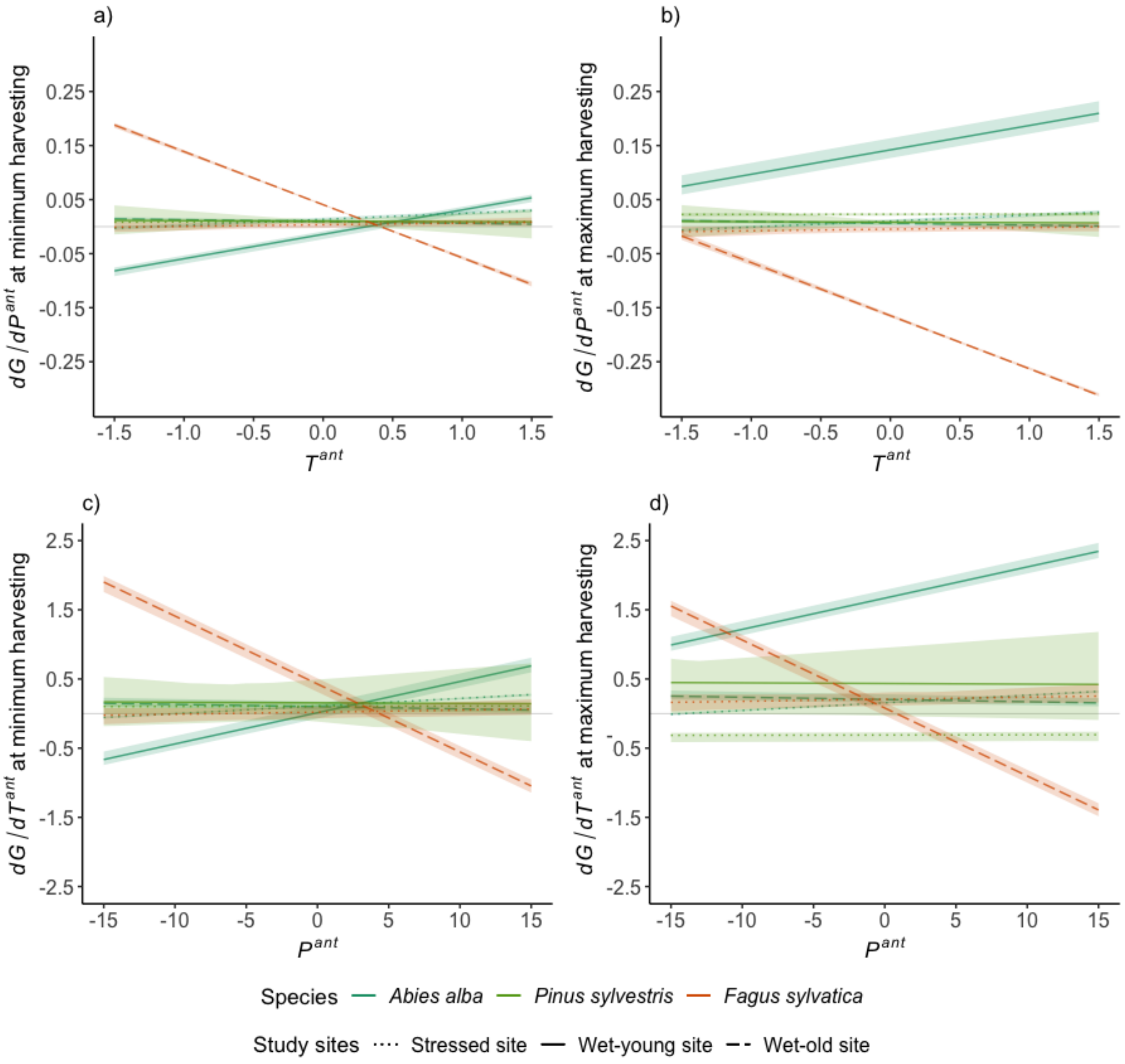

Appendix S10. Estimates of the net sensitivity of tree growth (*G*, see Eqn 1) to changes in P^{ant} (a, b) and T^{ant} (c, d), under minimum (a, c) and maximum (b, d) values of harvesting intensity in terms of wood volume removed from the stand (*HI*) for the subset of tree species and study sites for which models converged.

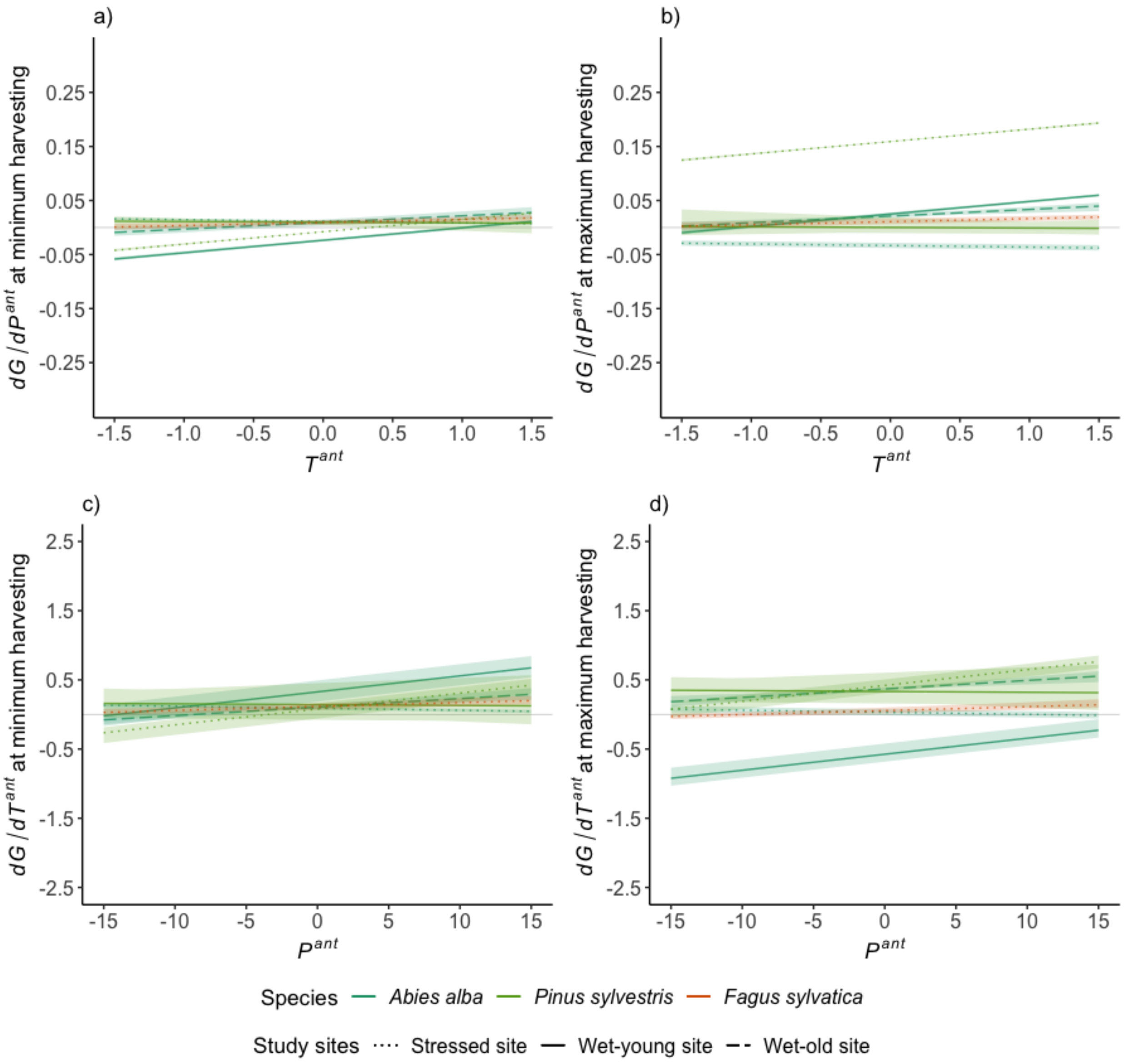

Supplement: Supplementary file 1 — (PDF 1614 KB) [file 10021_2021_650_MOESM1_ESM.pdf]
